# Pangenome Reveals Gene Content Variations and Structural Variants Contributing to Pig Characteristics

**DOI:** 10.1093/gpbjnl/qzae081

**Published:** 2024-11-13

**Authors:** Heng Du, Yue Zhuo, Shiyu Lu, Wanying Li, Lei Zhou, Feizhou Sun, Gang Liu, Jian-Feng Liu

**Affiliations:** State Key Laboratory of Animal Biotech Breeding, College of Animal Science and Technology, China Agricultural University, Beijing 100193, China; State Key Laboratory of Animal Biotech Breeding, College of Animal Science and Technology, China Agricultural University, Beijing 100193, China; State Key Laboratory of Animal Biotech Breeding, College of Animal Science and Technology, China Agricultural University, Beijing 100193, China; State Key Laboratory of Animal Biotech Breeding, College of Animal Science and Technology, China Agricultural University, Beijing 100193, China; State Key Laboratory of Animal Biotech Breeding, College of Animal Science and Technology, China Agricultural University, Beijing 100193, China; National Animal Husbandry Service, Beijing 100125, China; National Animal Husbandry Service, Beijing 100125, China; State Key Laboratory of Animal Biotech Breeding, College of Animal Science and Technology, China Agricultural University, Beijing 100193, China

**Keywords:** Pig, Pangenome, Heritability estimation, Genome-wide association study, Selection

## Abstract

Pigs are one of the most essential sources of high-quality proteins in human diets. Structural variants (SVs) are a major source of genetic variants associated with diverse traits and evolutionary events. However, the current linear reference genome of pigs restricts the accurate presentation of position information for SVs. In this study, we generated a pangenome of pigs and a genome variation map of 599 deeply sequenced genomes across Eurasia. Additionally, we established a section-wide gene repertoire, revealing that core genes are more evolutionarily conserved than variable genes. Furthermore, we identified 546,137 SVs, their enrichment regions, and relationships with genomic features and found significant divergence across Eurasian pigs. More importantly, the pangenome-detected SVs could complement heritability estimates and genome-wide association studies based only on single nucleotide polymorphisms. Among the SVs shaped by selection, we identified an insertion in the promoter region of the *TBX19* gene, which may be related to the development, growth, and timidity traits of Asian pigs and may affect the gene expression. The constructed pig pangenome and the identified SVs in this study provide rich resources for future functional genomic research on pigs.

## Introduction

As one of the most important livestock animals, pigs have a long shared history with humans and have sustained billions of people as a primary source of protein [1]. In addition to the traditional roles of pigs, a paradigm shift has occurred in recent years, with pigs emerging as an indispensable biomedical model owing to their profound anatomical, genetic, and physiological similarities to humans [2,3]. The publication of the pig genome contributed significantly to dissecting the genetic basis of distinct phenotypes and understanding the evolutionary processes of porcine domestication [2]. Although considerable advancements have been made to the Sscrofa11.1 assembly, which spans 2.5 Gb with 93 gaps, predominantly on chromosome Y, it falls short of encapsulating the full spectrum of genetic diversity across global pig breeds [4]. Despite ongoing refinement efforts, the reliance on a solitary female Duroc pig as the basis for this reference genome remains a limitation [5]. Comparative analyses of recently generated chromosome-level porcine assemblies have demonstrated substantial genetic variations among diverse breeds, particularly structural variants (SVs) [6]. An increasing number of studies have indicated the pivotal roles played by SVs in genome evolution and genetic control of economic traits in domestic animals [7,8]. However, conventional linear references face challenges in detecting large SVs and accurately inferring the genotypes for each locus [9]. The pangenome concept has been proposed and iteratively refined to overcome this limitation, thus providing a transformative approach to addressing the limitations mentioned above.

Pangenome is a reference that incorporates genetic diversity across diverse populations within a species [10]. Unlike conventional references based on single individuals, the pangenome provides a holistic representation of the gene contents of a species [10]. A contemporary trend in pangenome studies involves the construction of graph-based pangenomes that offer enhanced versatility as references [11]. Graph-based pangenomes demonstrate superior efficacy in genotyping SVs using low-cost short-read sequencing technologies compared to linear reference genomes. This attribute facilitates the precise and efficient identification of genomic diversity within a species [12]. Recent advancements in pangenome research have led to the development of comprehensive references for human populations [13] and animals [14,15]. With the development of pangenome mapping, identification, and genotyping algorithms, an increasing number of graph-based pangenomes have been constructed, and their utility in elucidating the genetic basis of diverse phenotypes across breeds within a species has been demonstrated [16]. In pigs, a pangenome has been constructed through a comparative *de novo* assembly approach, which scrutinized 11 scaffold-level genomes from various pig breeds and augmented the existing Sscrofa11.1 reference with additional 72.5-Mb novel sequences that account for approximately 3% of the genome [17]. However, the complete gene set in pigs remains obscure, and the extent of SVs and their biological effects on specific traits remain underexplored.

Here, we presented the *de novo* assembly of three chromosomal-level genomes, which were seamlessly integrated into a comprehensive pig pangenome alongside existing assemblies. The gene content within this pangenome was delineated, which elucidated its core, softcore, dispensable, and private genomes as well as the cladistic differences among the member populations. We comprehensively investigated SVs using this pangenome, revealing distinct SV distributions across Eurasian pigs. In combination with heritability estimations, expression quantitative trait locus (eQTL) mapping, genome-wide association studies (GWAS), and selective sweep analyses, we explored the role of SVs in determining missing heritability, deciphering the molecular mechanisms underlying trait variation, and driving genetic diversification across breeds. The constructed pangenome and detected genetic variants provide valuable resources for future functional genomic studies on pigs and will accelerate variome-based breeding in swine.

## Results

### *De novo* assembly and annotation of domestic pig genomes

**Figure 1:**
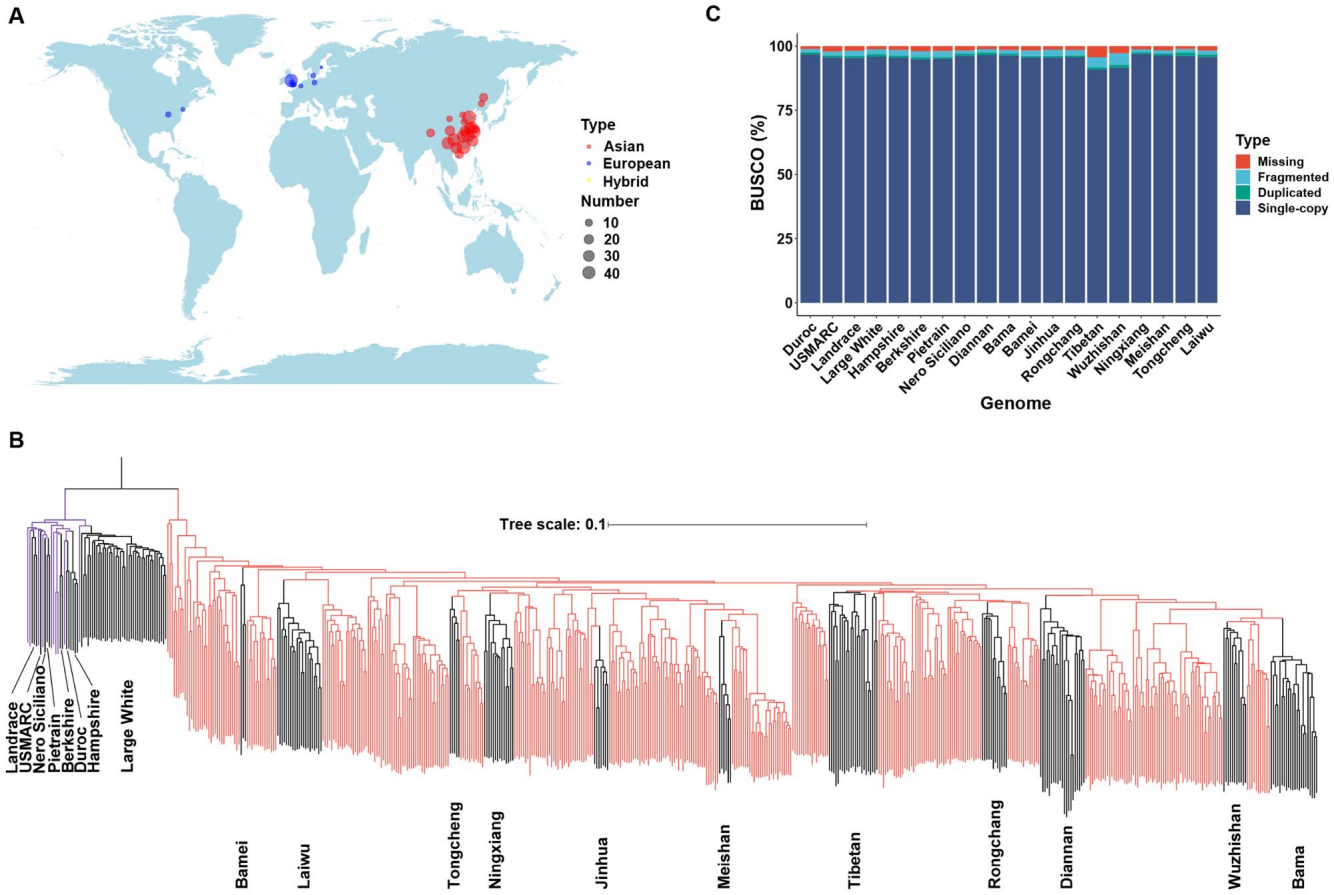
Geographic distribution and phylogenetic analysis of samples used in this study **A**. Geographic distribution of samples used in this study. The circle size represents the number of samples collected from each region, and the colors of the circles denote the origins of the samples (European pigs and pigs spread in America are indicated by blue, Asian pigs are indicated by red, and hybrid pigs are shown by yellow). The ggmap package in R generates this world map, and the map data are also achieved from this package. **B**. Phylogenetic tree of all samples constructed based on the whole-genome SNPs. Branches were color-coded to indicate European pigs (blue) and Asian pigs (red). The black branches within the European and Asian subclusters represent the three breeds selected for *de novo* genome assembly in this study and 16 breeds whose genomes have been assembled previously. **C**. Genome completeness of 19 assemblies assessed by BUSCO. SNP, single nucleotide polymorphism; BUSCO, Benchmarking Universal Single-Copy Orthologs.

To ensure that the pangenome represents the comprehensive genetic diversity of domestic pigs, we collected sequence data from 599 domestic pigs (525 were sequenced in our previous study [18], and 74 were downloaded from the public database), with an average coverage depth of more than 20× (Table S1). These 599 individuals represented a worldwide distribution of domestic pigs and consisted of 46 Asian indigenous pig populations, 14 European domestic porcine breeds, 2 breeds distributed in America, and a hybrid breed (**Figure 1**A). After aligning these sequence reads to the Sscrofa11.1 genome, 51,858,536 single nucleotide polymorphisms (SNPs) were identified.

Based on these SNPs, the phylogenetic tree classified the 599 individuals into two large subpopulations, distinctly characterized as Asian and European pigs. Notably, according to a previous investigation, the two American breeds and one hybrid breed were inclusively categorized as European pigs [19]. This taxonomic stratification aligns with previous findings, which suggest that the pig species (*Sus scrofa*) diverged into Asian and European lineages approximately 1.2 million years ago (MYA) [20]. Additionally, we discovered that Asian pigs could be classified further into four groups: BL subgroup (Bamei and Laiwu pigs), TNJM subgroup (Tongcheng, Ningxiang, Jinhua, and Meishan pigs), RT subgroup (Rongchang pigs and Tibetan wild boars), and DWB subgroup (Diannan Small-ear, Wuzhishan, and Bama Xiang pigs) (Figure 1B, Figure S1). To construct a representative pangenome, 15 genomes meeting the Benchmarking Universal Single-Copy Orthologs (BUSCO) criteria (> 90% completeness; Figure 1C; Table S2) [21] were judiciously selected, thus supplementing the Sscrofa11.1 genome. Three specific breeds were targeted for *de novo* assembly analysis to ensure a minimum of two assemblies per subgroup for robust pangenome exploration.

To capture the distinct traits exhibited by Chinese domestic pigs, characterized by high reproductivity, superior meat quality, and disease resistance, we employed the PacBio Sequel II platform to sequence three characteristic breeds: Meishan pig (noted for high reproductive capacity), Laiwu pig (renowned for superior meat quality), and Tongcheng pig (recognized for disease resistance, Table S3). This effort yielded a robust dataset comprising 315.45–355.51 Gb of data for each individual, with an estimated coverage depth of 126×–142× for different genomes. The assembled contig N50 values spanned from 10.93 to 17.90 Mb (Table S4). The contigs of these three genomes were corrected using corresponding deep whole-genome sequencing data. These corrected contigs were ordered, oriented, and clustered into 20 chromosomes using Hi-C data (Figures S2 and S3). Finally, we obtained chromosome-level genomes of the three breeds with a scaffold N50 of 137.13–140.06 Mb and genome length of 2.50–2.60 Gb. We further evaluated the completeness of the assembled genomes and found an average completeness of 97.13% (96.8%–97.5%) (Figure 1C). Moreover, we validated the completeness and accuracy of the three newly assembled genomes by aligning short reads from the same breed against each genome. The alignment results revealed an average mapping ratio of 99.59% for each genome. Simultaneously, an average of 95.22% of the genomic regions across each assembly exhibited a mapping coverage of at least 10×, attesting to the high continuity and completeness of the three assembled genomes.

The three new Chinese domestic pig genomes were annotated using a strategy combining genomic, transcriptomic, and protein-derived evidence. This integrative approach yielded an average of 22,675 protein-coding gene models in the three genomes. These models exhibited an average of 9 exons per gene and an average coding sequence (CDS) length of 166 bp (Table S5). The assessment of these gene models through BUSCO analysis revealed a high level of completeness, with an average of 92.5% for the 3354 single-copy vertebrate genes (Table S6). Additionally, an average of 93.30% of the annotated genes demonstrated the potential for functional allocation to at least one of the following six databases: GO_Annotation, KEGG_Annotation, KOG_Annotation, Swiss-Prot_Annotation, TrEMBL_Annotation, and NR_Annotation (Table S7). These results revealed the high completeness of the gene annotation.

### Gene-based pangenome of pig

**Figure 2:**
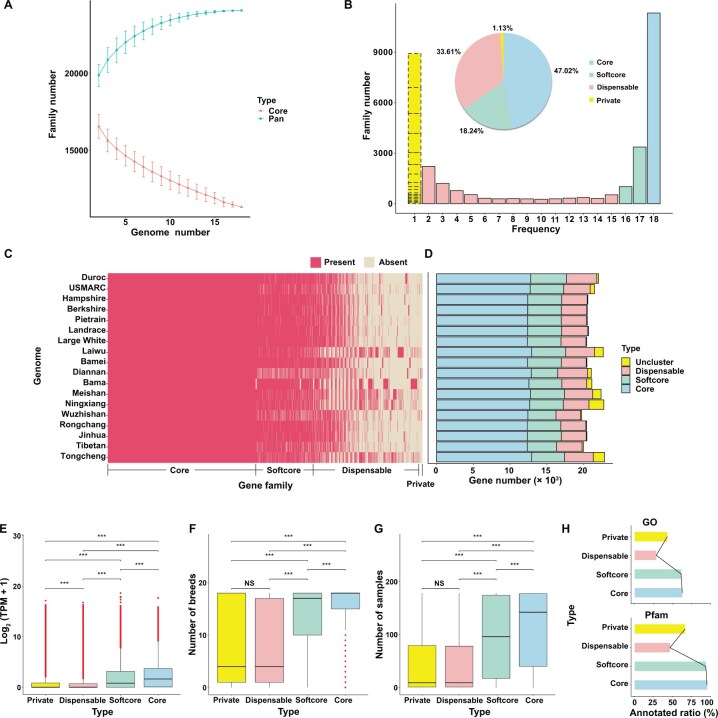
Pangenome and coregenome analyses of pigs **A**. Variation of gene families in the pangenome and coregenome when incorporating additional genomes into the pig pangenome. **B**. Composition of pan and core gene families. The bar plot shows the number of gene families with different frequencies. The yellow bar with a dashed border represents the number of unclustered genes within each genome. The pie chart indicates the proportion of different gene categories. **C**. Presence and absence of gene families in each genome. **D**. Gene number of different gene categories in individual genomes. **E**. Expression profiles of pan and core genes in Sscrofa11.1. **F**. Breed-specific expression profiles of pan and core genes in Sscrofa11.1. **G**. Expression width profiles (indicated by expressed or not in 171 RNA-seq data) of pan and core genes in Sscrofa11.1. **H**. GO and Pfam annotations for the core, softcore, dispensable, and private genes. ***, *P* < 0.001; NS, not significant (*t*-test). RNA-seq, RNA sequencing; GO, Gene Ontology.

After acquiring the three *de novo* genomes and integrating them with 15 previously downloaded genomes [excluding Nero Siciliano due to the absence of RNA sequencing (RNA-seq) data and inadequate annotation], pangenome analyses were conducted using a previously reported strategy [22]. Based on an analysis of the orthologs, all genes across the 18 pig genomes were categorized into 24,087 families. The cumulative gene sets exhibited an incremental pattern, reaching a plateau at *n* = 15 genomes (**Figure 2**A), indicating the representativeness of the 18 pig genomes. Among the total gene family set, 11,326 were detected in all 18 pig genomes and were defined as the core gene families (47.02%). Additionally, 4394 families (18.24%) present in 16–17 genomes were identified as softcore gene families, 8095 families (33.61%) detected in 2–15 genomes were categorized as dispensable gene families, and 272 families (1.13%) identified in only one genome were classified as private gene families (Figure 2B). Consistent with a previous study [22], the percentage of core genes declined with the addition of pig genomes, although it did not reach a trough even at *n* = 18 (Figure 2A). Furthermore, the total number of core and softcore gene families accounted for more than half (65.26%) of the gene family set in the 18 genomes (Figure 2C). In each genome, these families accounted for an average of 80.75% of the protein-coding genes (Figure 2D). However, each genome still had an average of 17.01% dispensable gene families. These observations indicated that each pig genome contained a subset of genes that were dominant in the individual gene set and possessed a significant number of unshared genes.

We further investigated the expression profiles of pan genes using RNA-seq data from 171 samples of 18 breeds (Table S8). The overall expression level, breed-specific expression status (expressed or not in the 18 breeds), and expression width (expressed or not in the 171 samples) gradually decreased from the core, softcore, dispensable, and private genes (Figure 2E–G). These results suggested that the core genes exhibited a steady and broader expression spectrum, while the variable genes showed lower, tissue-specific, breed-specific expression. Furthermore, we discovered that approximately 95.46% of the core genes and 93.83% of the softcore genes contained protein domains (reflected by Pfam), and these values were higher than the proportions of dispensable and private genes (46.52% and 66.54%, respectively) (Figure 2H). Gene Ontology (GO) annotation revealed similar results (Figure 2H), indicating that the core genes were more evolutionarily conserved than dispensable and private genes.

### Large genomic variations and hotspot regions

**Figure 3:**
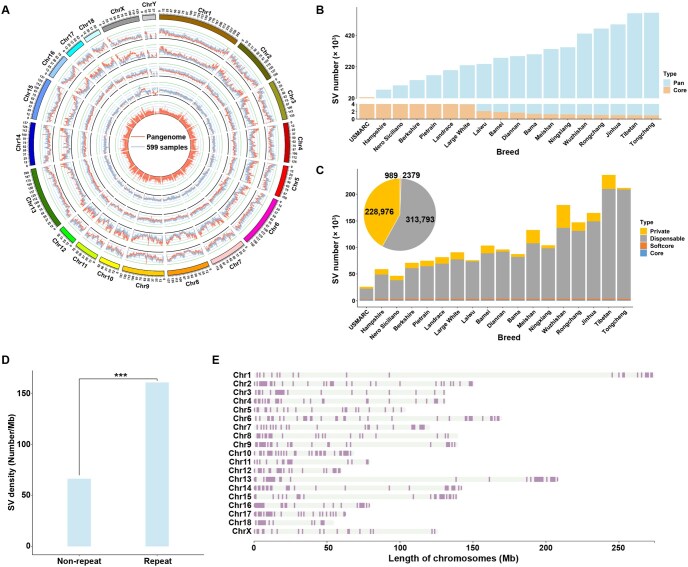
Genetic variations from 18 assemblies and 599 whole-genome sequencing individuals **A**. Distribution of genetic variations. Genetic variations were analyzed using 18 assemblies (compared to the Sscrofa11.1 reference genome) and 599 whole-genome sequencing individuals. The Circos plot shows the following features (from outer to inner): ideogram of the porcine genome with colored karyotype bands, SNP density, nucleotide diversity (π), transition/transversion ratio (Ts/Tv), Tajima’s *D*, and SV density across the 18 genomes. **B**. Non-redundant merging of SVs. SVs detected in each genome were merged using a non-redundant strategy, starting with the USMARC genome and iteratively incorporating new SVs from additional genomes. **C**. Distribution of SVs. The bar plot indicates the number of SVs of different SV categories in each genome. The pie chart shows the proportion of different SV categories. **D**. SV density in the repeat and non-repeat genome regions. ***, *P* < 0.001 (*t*-test). **E**. Distribution of the 495 SV hotspots in the porcine genome. SV, structural variant.

We aligned all 18 assemblies to the Sscrofa11.1 genome to detect genomic variants. We identified 41,724,488 SNPs in the 18 pig genomes relative to the reference Sscrofa11.1 genome (Figure S4). Although the number of SNPs from the 18 assemblies was lower than that from the 599 individuals (41,724,488 *vs*. 51,858,536), the distributions of SNPs from these two datasets exhibited similar patterns across the genome (**Figure 3**A). The correlation between SNP distributions from the 18 pig assemblies and 599 individual genomes was notably high at 0.91 (Pearson’s correlation test, *P* < 0.05; Figure S5A). Moreover, we estimated the nucleotide diversity (π), transition/transversion ratio (Ts/Tv), and Tajima’s *D* value in the 18 assemblies and 599 individual genomes (Figure S5B–D). The distributions of these features across the porcine genome exhibited high correlations between the 18 assemblies and 599 individual genomes, further indicating the representativeness of these 18 assemblies for Eurasian boars.

In addition to SNPs, the assembled genomes allowed us to identify large SVs (> 50 bp in this study) by comparative genomic analysis between different breeds. We compared the 18 assemblies with the Sscrofa11.1 assembly and identified three types of SVs: presence/absence variations (PAVs), inversions, and translocations. An average of 71,704 SVs per genome were determined relative to Sscrofa11.1, and they spanned 68.70 Mb of genomic regions per breed on average. Subsequently, we merged the SVs across the 18 genomes into a set of non-redundant SVs. A total of 546,137 SVs made up of 772.92-Mb genomic regions were obtained, including 541,033 PAVs, 1887 inversions, and 3217 translocations (comprising 2231 intra-chromosome translocations and 986 inter-chromosome translocations). We discovered that most PAVs ranged from 1 to 2 kb, inversions mainly ranged from 10 to 20 kb, and translocations varied from 2 to 3 kb (Figure S6). Simultaneously, the non-redundant SV set grew and tended to flatten as more assemblies were added (Figure 3B). In contrast, the set of shared SVs declined, leaving a total of 989 SVs that were shared in all assemblies (Figure 3B).

We categorized these SVs into four classes (Figure 3C) according to their frequencies in the 18 assemblies: core (present in all 18 genomes), softcore (present in > 90% of genomes but not all), dispensable (present in more than one genome yet < 90% of genomes), and private (only detected in one genome). We found that Asian indigenous pigs showed higher dispensable SVs (average of 122,106; *t*-test, *P* = 6.11 × 10^−4^) than European domestic pigs (average of 51,644) (Figure 3C). However, the number of private SVs in different Asian indigenous pigs exhibited more extensive diversity [coefficient of variation (CV) = 0.87] than that in European pigs (CV = 0.34). We found that SVs tended to be enriched in repetitive DNA regions (Figure 3D). We investigated the sequence composition of each PAV. We found that 36.75% of the PAVs originated from repetitive DNA, which supports the insight that variations in repetitive sequences might contribute to the divergence of distinct genomes.

Our examination of SVs arrayed across chromosomes revealed an uneven distribution along the chromosomes, indicating that multiple and independent SVs arose in these regions. In total, we identified 495 hotspots (Figure 3E; Table S9) that overlapped with 2515 quantitative trait loci (QTLs), which were enriched in pathogen and parasite traits (*P* = 0.012). Furthermore, we discovered an uneven distribution of SV hotspots between distinct porcine subpopulations. In this study, we detected 2677 putative SV hotspots across five different populations (the European pig population and four distinct Asian pig populations) (Figure S7). Notably, we found 47 SV hotspots enriched over a 50.70-Mb region on chromosome X (49.80–100.50 Mb) and 20 SV hotspots enriched over a 25.80-Mb region on chromosome 5 (70.40–96.20 Mb) in four Asian indigenous populations; however, such enrichment was not observed in European pigs (Figure S7). In particular, the SV hotspot enrichment region on chromosome X overlapped with a previously reported region (45–87 Mb) and contained different haplotypes between Asian and European pigs [23]. In addition, we noticed an extended region of 42 Mb (70–112 Mb) on chromosome 9 that only contained an SV hotspot in European pigs. Notably, this hotspot region showed 41 independent SVs and resided in an important *CRPPA* gene that plays a critical role in skeletal muscle development, structure, and function [24].

### Impact of SVs on functional genomic regions

**Figure 4:**
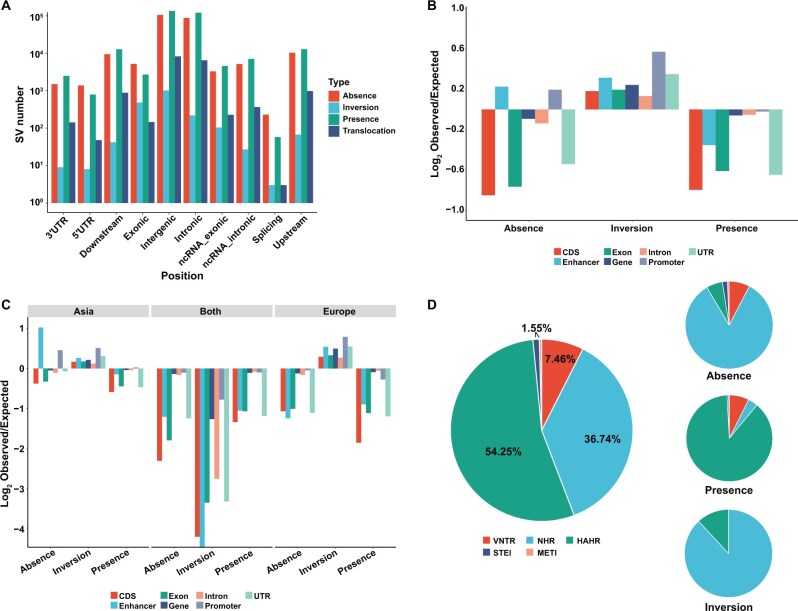
Impact of SVs on functional genomic regions and their formation mechanisms **A**. Distribution of different SV types across various genomic regions. **B**. Enrichment/depletion of different SV types within seven functional genomic regions. **C**. Enrichment/depletion of different SV classes in European and Asian pigs within seven functional genomic regions, respectively. **D**. The proportion of SVs formed by the five estimated formation mechanisms in pigs. UTR, untranslated region; CDS, coding sequence; ncRNA, non-coding RNA; VNTR, variable number of tandem repeat; NHR, nonhomologous recombination; NAHR, non-allelic homologous recombination; STEI, single transposable element insertion; MTEI, multiple transposable element insertion.

As SVs significantly influence genomes and are often associated with specific traits, we systematically evaluated the possible functional effects of SVs on both coding and non-coding regions. First, we discovered that most SVs were located in intergenic regions (**Figure 4**A, Figure S8). Almost half of the SVs (47.51%) were associated with at least one gene, with 43.78%, 6.83%, and 1.52% found within protein-coding genes, long non-coding RNA genes, and pseudogenes, respectively. Furthermore, we investigated the associations between the SVs identified in this study and the seven distinct genomic features annotated: gene, exon, intron, CDS, promoter, untranslated region (UTR), and enhancer. Enrichment analysis of these features revealed that most SVs were depleted in the genic areas, specifically in CDSs (Figure 4B; File S1). However, the inversion did not follow a similar depletion trend, which demonstrated enrichment across all seven features, possibly caused by the large size of most inversions. We found that 16.75% of the inversions contained an average of 8 whole genes, whereas only 0.29% of the absences contained at least one whole gene.

Notably, absences occurred more frequently than presences in the promoter and enhancer regions. These SVs were divided into three categories: (1) SV exclusively present in European pigs; (2) SV detected only in Asian pigs; and (3) SV occurring in both groups. We conducted a comprehensive analysis to gain deeper insights into the relationship between genomic features and the three categories of SVs (Figure 4C). The results showed that the presence or absence of these three categories resulted in similar depletion signatures for genomic features, except for promoters and enhancers. In particular, the absence and presence of promoters in Asian pigs indicated enrichment of this genomic feature, which may correspond to the characteristics of Asian pigs.

### Inferring the mechanisms of SV formation

Discovering the specific mechanisms and driving forces of SV formation could improve our understanding of these large genomic variations and facilitate further studies on how large variants affect phenotypes. In this study, we employed a validated workflow [25] based on an analysis of breakpoint junction sequences of SVs to infer the mechanisms of SV formation.

In total, 91.39% of the detected SVs (excluding translocations) were categorized into five types based on their inferred formation mechanisms. Among the assigned SVs, non-allelic homologous recombination (NAHR) (54.25%) was the dominant formation mechanism, followed by nonhomologous recombination (NHR) (36.74%) (Figure 4D). The observed formation percentage differed from the primary mechanism in human genomes, in which NHR is the dominant SV formation mechanism [25]. Apart from the abovementioned dominant SV formation mechanisms in pigs, our analysis indicated that approximately 44,700 SVs were shaped by variable number of tandem repeat (VNTR) and transposable element insertion (TEI) (Figure 4D).

In addition, a similar investigation revealed that the dominant SV formation mechanism varied among the different types of structural variations (Figure 4D). For instance, most presences originated from NAHR (88.09%), followed by VNTR (7.38%). However, absences and inversions exhibited distinct patterns shaped primarily by NHR. VNTR was the second most dominant SV formation mechanism among absences (7.64%), whereas NAHR was the second most dominant SV formation mechanism among inversions (11.92%). In addition to the SV type, we examined the dominant SV formation mechanism in different SV length categories. The results showed that almost half of SVs shorter than 100 kb were formed by NAHR, while SVs longer than 100 kb were mainly formed by NHR. In addition, concentrating on the two categories of TEI, we noticed that the length of SVs that originated from a single transposable element insertion (STEI) was mainly distributed from approximately 200 to 500 bp. In contrast, the length of SVs formed by multiple transposable element insertion (MTEI) was primarily centered at approximately 5–10 kb.

### Pangenome graph utility in pigs

**Figure 5:**
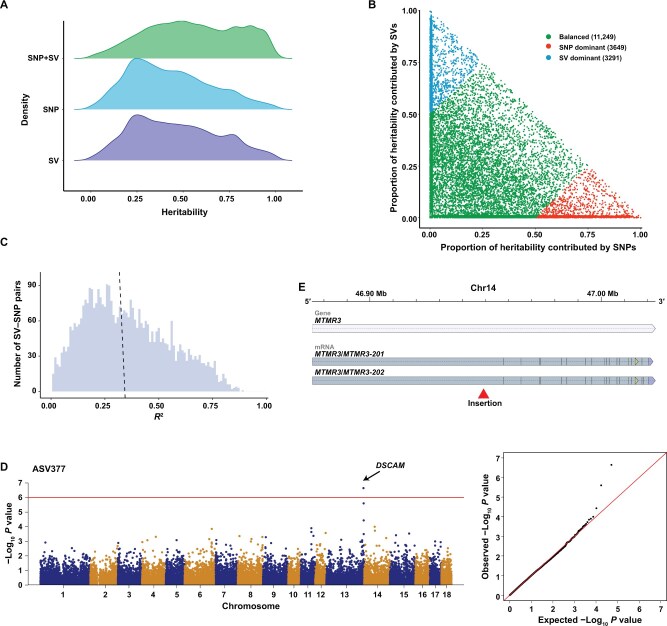
Utility of the graph-based pangenome **A**. Comparison of heritability estimated using different categories of genetic variants. **B**. Contribution of SNPs and SVs to trait heritability. **C**. LD distribution between eQTLs detected by SVs and SNPs for the same genes. The dashed line represents the average LD value. **D**. SV-based GWAS result for ASV337. The left is the Manhattan plot, and the right is the quantile-quantile plot. **E**. The gene structure of *MTMR3* and the position of the insertion in its intron. The horizontal blue bar represents the gene region, and the horizontal gray bars indicate the transcripts. The red arrow marks the breakpoint of the insertion. LD, linkage disequilibrium; eQTL, expression quantitative trait locus; GWAS, genome-wide association study.

We built a graph-based pangenome using Sscrofa11.1 as a linear base reference and integrated non-redundant PAVs without many repetitive sequences (ratio < 90%). In total, 353,702 PAVs were integrated into the graph-based pangenome (Figure S9). We have previously reported data on 300 European-Asian hybrids [26], including genomic, transcriptomic, and microbiomic (sampled from the feces, cecum content, ileal content, ileal mucosa, and cecum mucosa) data, which allowed us to investigate whether the pangenome graph could detect SVs that lead to phenotypic variations in any traits. To test the power of the graph-based pangenome in capturing missing heritability, we used the LDAK [27] method to estimate the heritability of 18,189 molecular traits, including 16,037 expression traits and 2152 microbiota traits. After quality control, 286,571 SNPs and 25,933 SVs were retained for subsequent analyses (see Materials and methods). We analyzed the contribution of these genetic variants to molecular traits individually (SNPs or SVs) and jointly (SNPs + SVs). The average heritability estimated using SVs was higher than that using SNPs (0.46 *vs*. 0.43; Wilcoxon rank sum test, *P* = 3.42 × 10^−82^). Heritability estimates increased when both SVs and SNP categories were incorporated into the model (**Figure 5**A). The estimated heritability of genetic variants jointly was significantly higher than that of SNPs alone (0.56 *vs*. 0.43; Wilcoxon rank sum test, *P* < 1 × 10^−230^) or SVs alone (0.56 *vs*. 0.46; Wilcoxon rank sum test, *P* < 1 × 10^−230^). Furthermore, although both SNPs and SVs balanced the heritability of most molecular traits, traits predominantly explained by SVs still existed (Figure 5B; File S1). These results indicate that SVs identified using the pangenome approach can help capture missing heritability.

In addition, we detected *cis*-eQTLs based on the SNPs and SVs. Among 3137 genes, *cis*-eQTLs were detected using both SNPs and SVs. Notably, the linkage disequilibrium (LD) between *cis*-eQTLs, determined separately based on SNPs and SVs for each gene, was low (average *R*^2^ = 0.34). Only a small proportion of SVs (0.96%) were in high LD (*R*^2^ > 0.8) with the SNPs (Figure 5C). This result indicates that low LD between different marker types is common in this public population. In addition, 139 expressed genes in this population were detected in eQTLs based on SVs but not on SNPs; this could partially indicate that the SVs detected using the graph-based pangenome assist in recognizing causal variants.

Furthermore, we performed a GWAS based on SVs to explore the correlation between SVs and the gut microbiota composition in the public population. The 16S rRNA tags (V3–V4) were clustered into amplicon sequence variants (ASVs). In the fecal samples, we identified two lead SVs associated with ASV377 and ASV2205 (Figure 5D, Figure S10). In particular, for ASV377 assigned to the Bacteroidales order (Table S10), we identified a tag-SV located in the intron of the *DSCAM* gene that was associated with its abundance. This gene has been implicated in immune specificity and memory [28], and the products of this gene are particularly effective against invading parasitic or bacterial pathogens and can even be related to gut microbiota management [29].

In addition, the graph-based pangenome provided an opportunity to detect important functional genes. In our 599 genomes, 932 tag-SNPs from a previous GWAS atlas [30] were obtained and used to search for SVs with strong LD with these tag-SNPs. In total, 102 SVs with high LD (LD > 0.8) were discovered, of which 36 overlapped with the introns of 26 distinct genes (Table S11). Among these genes, the *MTMR3* gene drew our attention. Previous studies have reported an important role of *MTMR3* in the proliferation and differentiation of skeletal muscle satellite cells [31]. The insertion in the intron of the *MTMR3* gene (Figure 5E) showed high LD with the tag-SNP (NC_010456.5:g.47895001C>T) for the gestation length phenotype. This insertion may also be considered a marker for the gestation length phenotype, and the *MTMR3* gene could be an important gene for fetal development in utero [32].

### Contribution of SVs to the diversification of Eurasian pigs

**Figure 6:**
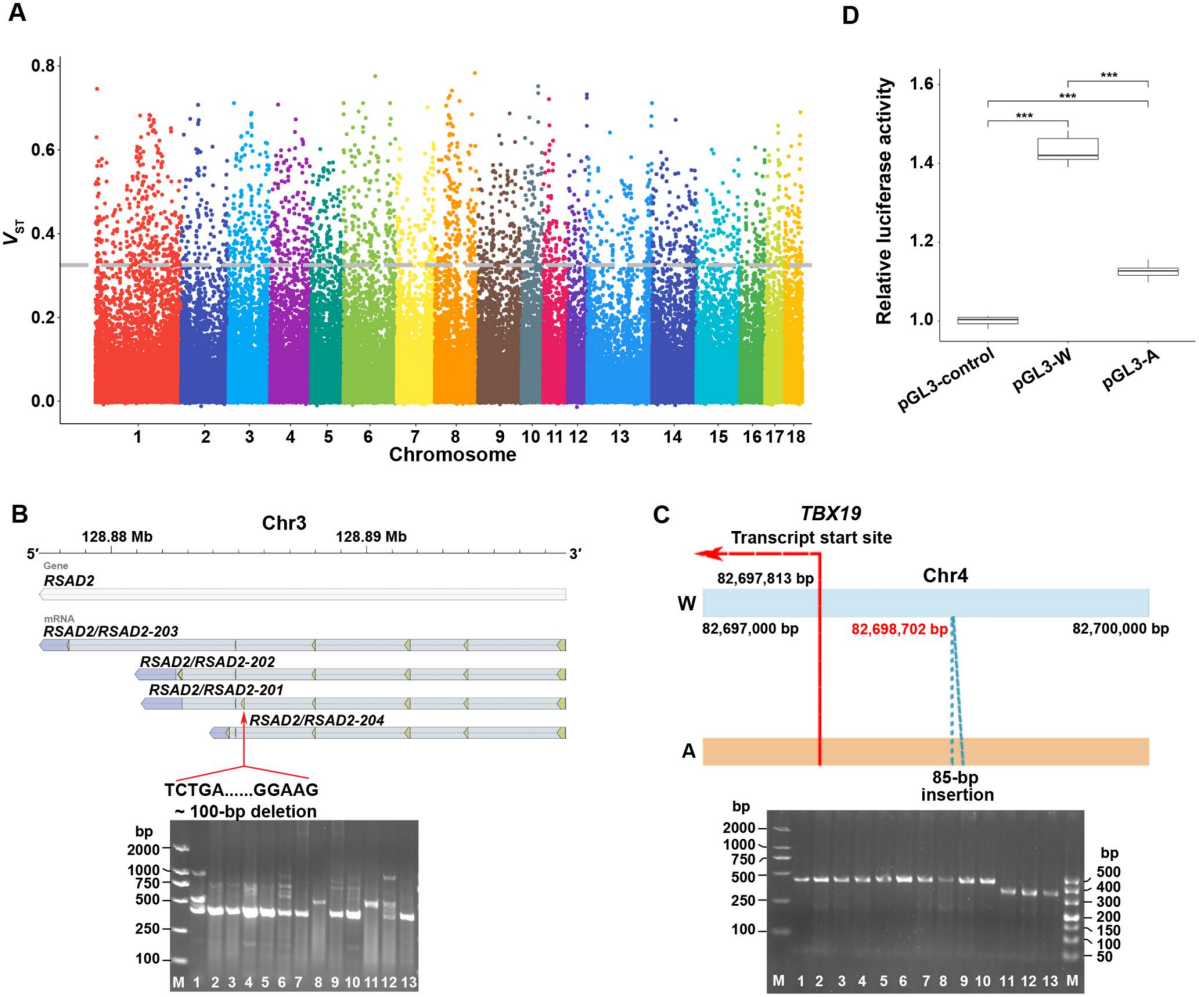
Genome-wide screening and selective signatures between European and Asian pigs **A**. Manhattan plot showing the *V*_ST_ values calculated for selected SVs through pairwise comparison with a threshold *V*_ST_ value ≥ 0.33. **B**. The gene structure of *RSAD2* and the position of the deletion in its exon. The image below shows the PCR results for detecting the presence or absence of this deletion in 13 breeds. The numbers from 1 to 13 represent the breeds as follows: Wuzhishan pig, Tongcheng pig, Bama Xiang pig, Rongchang pig, Ningxiang pig, Meishan pig, Diannan Small-ear pig, Bamei pig, Jinhua pig, Tibetan wild boar, Large White pig, Landrace, and Duroc. M denotes the DNA markers used. **C**. Allele variation in the promoter region of *TBX19* gene. The blue horizontal bar represents the wild-type (W) allele, and the orange horizontal bar represents the allele with an 85-bp insertion in the promoter (A). The blue dashed lines between the two horizontal bars indicate the position of the insertion. The image below shows the PCR results for detecting the presence or absence of the insertion in 13 breeds. The numbers from 1 to 13 represent the same breeds as in (B). **D**. Comparison of transcriptional activities of different *TBX19* promoter variants using a luciferase reporter assay in 293T cells. ***, *P* < 0.001 (*t*-test).

Numerous genetic loci have undergone selection throughout the speciation process in European and Asian pigs, and many studies have suggested this phenomenon based on SNP data. In this study, we genotyped SVs in the 599 porcine genomes across Eurasia using our constructed graph-based pangenome. We calculated a validated statistical *V*_ST_ [33] to identify significant and genetically differentiated SVs within populations. A total of 2424 SVs representing the top 1% of *V*_ST_ values were identified, suggesting potential selective signatures (**Figure 6**A). Among these SVs, 784 genes overlapped, and functional enrichment analysis of these genes revealed enrichment in functions related to disease resistance, energy metabolism, and other relevant pathways (Table S12). These genes were within 1005 QTLs referencing 25 phenotypes (Table S13). Furthermore, we concentrated on genes whose CDSs were affected by these potentially selected SVs (Table S14). Finally, nine genes were detected, among which *RSAD2* plays a critical role in immune response. A ∼ 100-bp deletion was identified, overlapping with the fifth exon of the *RSAD2* gene. Polymerase chain reaction (PCR) analysis in various randomly selected breeds further validated this deletion (Figure 6B). Furthermore, we aligned the RNA-seq data of Asian and European pigs to Sscrofa11.1 and validated the existence of this deletion (File S1; Figure S11). These alignment results indicated that this deletion induced a new splice junction in the *RSAD2* gene and might influence its expression.

Previous enrichment analyses revealed the enrichment of SVs within promoter regions; thus, we also endeavored to discover genes with promoters under selection. A total of 35 genes were identified, and 9 were enriched in GO terms related to the cytosol (Table S15). We also performed an SNP-based selective sweep analysis on the same populations using the fixation index (*F*_ST_) and nucleotide diversity (π) methods (Figure S12). Enrichment analysis suggested that the SVs under selection showed significant enrichment in SNP-based selective sweep regions compared to the random background model (*P* < 0.01, Figure S12). In total, 62 SVs were under potential selection within the significant selective sweep regions detected by the SNPs. More importantly, an SV in the promoter of *TBX19* was under significant selection based on both SV and SNP detections. To further dissect the function of this SV, we first analyzed the PCR results for this SV breakpoint and identified two alleles: the wild-type allele (W) and an allele with an 85-bp insertion in the promoter of *TBX19* (A) (Figure 6C). To further assess the molecular effects of this insertion, luciferase expression levels were measured to determine the transcriptional activity by transfecting two types of recombinant plasmids (pGL3-A and pGL3-W) into 293T cells. Before performing the luciferase activity assay, we screened the genome region that contained the pGL3 construct and confirmed that no differences occurred except for the A insertion. Therefore, the difference in activity between the two constructs was due to the insertion. The activities of the pGL3-A and pGL3-W groups were higher than those of the pGL3-control group (*t*-test). Moreover, the transcriptional activity of the insertion allele (A) was significantly lower than that of the wild-type allele (W) (*t*-test, *P* = 3.73 × 10^−5^; Figure 6D). These results suggest that this 85-bp insertion in the promoter of *TBX19* may regulate its expression.

### Differences in the Y chromosome between European and Asian boars

**Figure 7:**
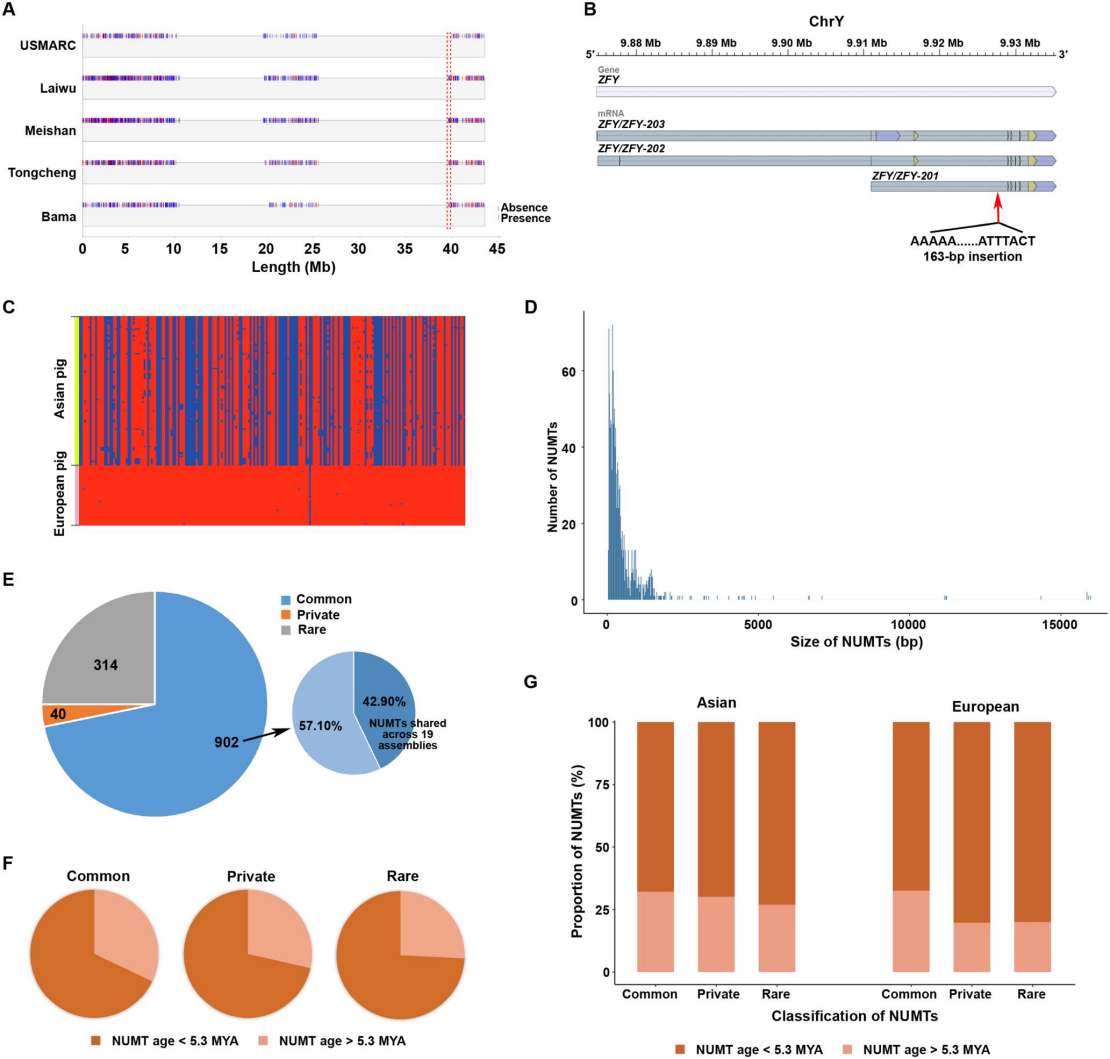
SVs detected in the Y chromosome and distribution of NUMTs in the porcine genome **A**. SVs detected in the Y chromosome using long-read sequencing data from five boars. The red dashed rectangle indicates the regions where SVs were present in the four Asian breeds but absent in the European breed. **B**. The gene structure of *ZFY* and the position of the insertion in its intron. **C**. SNP distribution in the *ZFY* gene region across the Eurasian pigs. Blue blocks represent the presence of SNPs, while red blocks indicate their absence. **D**. Size distribution of NUMTs. **E**. Left: pie chart showing the number of NUMTs in three different categories. Right: pie chart showing the proportion of NUMTs detected in all 19 assemblies or with frequency > 0.1 in all assemblies. **F**. Pie charts showing the proportion of older and younger NUMTs in the common, rare, and private categories. **G**. Box plot showing the proportion of older and younger NUMTs in three categories in European and Asian pigs, respectively. MYA, million years ago; NUMT, nuclear mitochondrial DNA segment.

Our study provides long-read sequencing reads from three male pigs and offers an opportunity to discover differences between European and Asian boars based on the Y chromosome. The Y chromosome of Sscrofa11.1 was used as the reference Y genome, and the long-read sequences of four Chinese domestic boars (Laiwu, Meishan, Tongcheng, and Bama Xiang) and one European hybrid boar (USMARC) were aligned to the reference. We finally detected 1485 SVs on the Y chromosome (**Figure 7**A). A total of 146 SVs were detected in genomes of all four Chinese domestic boars but absent in the genome of the European hybrid boar. Of these, 31 SVs were located in the pseudoautosomal region (PAR), whereas the remaining resided in the non-pseudoautosomal region (NPAR). Moreover, 77.40% of the 146 SVs were in intergenic regions. Notably, in the NPAR of the Y chromosome, we identified a new insertion in the intron of the *ZFY* gene (Figure 7B). Further analysis identified distinct haplotypes of this gene between European commercial pigs and Asian indigenous pigs (Figure 7C; File S1). We also identified a new insertion in the exon of the LOC100624149 gene (Figure S13), also known as *EIF2S3Y*, which has been previously reported to be associated with spermatogenesis [34].

In addition, in the Y chromosome, we identified a 394-kb region (39.56–39.95 Mb) containing SVs only found in Chinese domestic boars (Figure 7A). Comparative analysis of this region with other regions of the Y chromosome revealed an extremely low SNP density (0.04 variants/kb) compared to the average density (0.59 variants/kb). More importantly, most SNPs in this region (72.73%) were rare variants (< 0.05) and might have little influence on this genomic region. Among the 17 SVs detected in this region, five were present in all four Chinese domestic boars, including a ∼ 7-kb deletion in the intronic region [adjacent to the 5′ UTR (3.5 kb)] of the LOC110257970 gene (Figure S14; Table S16). The LOC110257970 gene contained only this deletion. This gene is a BCL-6 corepressor-like gene, and one of its conserved domains is homologous to the non-ankyrin-repeat region of the *BCOR* gene, which may be related to germinal center formation and apoptosis [35].

### Characterization of nuclear DNA of mitochondrial origin in the pig genome

We employed a validated nuclear mitochondrial DNA segment (NUMT) detection pipeline [36] to detect the NUMTs in the 19 genome assemblies. In total, 12,002 NUMTs were identified, with a median of 613 NUMTs per assembly, ranging in size from 34 to 15,975 bp. After removing redundant NUMTs, we achieved 1256 unique NUMTs across the 19 genomes (Figure S15). Most NUMTs were short insertions, with 50.80% less than 300 bp and 71.10% less than 500 bp (Figure 7D). We categorized the NUMTs into three classes (Figure 7E): common [frequency (*F*) ≥ 0.1], rare (*F* < 0.1), and private (detected only in one assembly). In total, 902 NUMTs (71.82%) were classified as common (including 387 shared across all genomes), 40 were rare, and 314 were private. By integrating NUMT data with the three newly assembled genomes in this study, we identified 29 NUMTs that, to our knowledge, have not been reported previously. We compared the 1256 NUMTs to the annotation of the Sscrofa11.1 reference genome and identified 95 germline NUMTs located in gene regions, with the majority (82.11%, *n* = 78) being enriched in introns against exons (Table S17). The NUMTs located in the CDSs of the reference genome were common (*F* ≥ 0.1) for the 19 assemblies, and these affected genes were all novel genes.

To understand the molecular evolution of NUMTs, we estimated when these insertions occurred in the Suinae lineage by comparing modern mitochondrial DNA (mtDNA) sequences with a consensus Suinae mtDNA sequence and detecting diagnostic sites that match specific positions in each NUMT. Finally, we estimated the ages of 657 NUMT insertions. The majority (69.71%) of NUMTs occurred less than 5.3 MYA, with 349 originating during the Pliocene period (2.5–5.3 MYA). As expected, common NUMTs were significantly enriched with older NUMTs compared to rare and private NUMTs (Figure 7F). Notably, the private and rare NUMTs in European pigs were younger than those in Asian pigs (Figure 7G). Overall, our results indicate ongoing NUMT insertion and evolution throughout pig evolutionary history.

## Discussion

Pigs are domesticated animals that play a vital role in providing sufficient protein resources to populations worldwide. A deeper understanding of the latent genomic variation underlying porcine diversity will significantly benefit the development of animal husbandry and provide resources for the broader biological and genomic research communities. In this study, we performed *de novo* assembly and annotation of three new male porcine genomes by integrating data from PacBio long-read sequencing, paired-end short-read sequencing, and Hi-C technologies. More importantly, we constructed a pangenome of the pig by assembling and comparatively analyzing the three new chromosomal-level genomes alongside 16 publicly available genomes representing diverse populations. Remarkably, our analyses detected a large number of genetic variants among the 18 genomes and Sscrofa11.1 reference assembly (an average of 10,215,593 SNPs, 71,704 SVs, and 68,538 PAVs) compared to previous SV studies based on short-read DNA sequencing data [37,38]. In addition, to limit contamination by new sequences, only non-reference sequences larger than 300 bp were retained from the previously published porcine pangenome based on 11 contig-level chromosomes; therefore, some variants might have been omitted. In this study, using the new chromosomal-level genomes, we detected 66.17 Mb of new sequences that complemented the previous pangenome. Furthermore, our enrichment analysis of SVs and genomic features revealed strong associations, particularly the frequent occurrence of absences around promoters or enhancers, thereby demonstrating the potential function of SVs in regulating gene expression. The newly constructed pangenome offers insights into genomic variation across Eurasian indigenous pigs and provides a valuable resource for functional genomic studies in pigs.

Indigenous Asian and European pigs are independently domesticated in Asia and Europe. Previous studies have reported that long-term independent domestication has left unique genomic footprints in Eurasian domestic pigs, such as selective sweeps of European variants around the *NR6A1*, *PLAG1*, and *LCORL* genes [39] and strong selective sweep signals at *GPR149* and *JMJD1C* in Tongcheng pigs [40]. In this study, we focused on the distribution of SV hotspots in European and Asian pigs. We discovered only a long SV hotspot cluster region in chromosome X (49.80–100.50 Mb) of Asian pigs, which aligns with a previously reported X-linked selective sweep region in Eurasian pigs [5], further confirming the differences between European and Asian pigs in this region. In addition, we detected a new region which contained an SV hotspot exclusive to European pigs and was located in *CRPPA*, an important gene vital for skeletal muscle development, structure, and function [24]. To understand the contribution of SVs to the divergence between European and Asian pigs, we employed SVs to identify the regions under selection in Asian pigs compared to European pigs. The selection regions identified by SVs tended to overlap with the selective sweep regions detected by SNPs in our enrichment analysis, albeit with only a modest number of overlapping windows. This result is consistent with previous SV studies in rice [41]. In the overlapping regions, we identified an SV residing in the promoter of the *TBX19* gene, which has been previously reported to be under selection based on SNP analyses [42,43]. *TBX19* is a protein-coding gene belonging to the T-box family of transcription factors, which play pivotal roles in regulating developmental processes [44]. Functionally, TBX19 serves as a positive regulator of pro-opiomelanocortin (*POMC*) expression, thereby influencing the production of adrenocorticotropic hormone (ACTH) within the hypothalamic-pituitary-adrenal (HPA) axis. Deficiency in ACTH is associated with symptoms of adrenal insufficiency, including weight loss, reduced appetite, obesity, hypoglycemia, and low blood pressure [45]. Previous studies have reported that *TBX19* is related to the development, growth, and timidity traits in Chinese native pigs [42,43]. Our luciferase reporter assay further demonstrated that the newly detected SV in the *TBX19* promoter has the potential to alter the expression of this gene. Furthermore, H3K27ac enhancer research validated the presence of the high-activity peak within the promoter of the *TBX19* gene in Bama Xiang pigs [46]. Overall, the SV identified under selection in the *TBX19* promoter appeared to have a regulatory effect on the expression of this gene, making it a promising candidate for further investigation into the developmental and timidity traits in Chinese domestic pigs.

In this study, our pangenome graph highlighted the importance of SVs in capturing missing heritability. In particular, with the inclusion of SVs, the estimated heritability significantly increased compared to that estimated using SNPs alone, consistent with the findings from a previous study [16]. In addition, we found that SVs contributed the largest share of heritability for ∼ 6.14% of molecular traits, indicating that the detected SVs could be excellent genetic markers to compensate for the difficulty of elucidating traits by SNPs. Additionally, numerous studies have demonstrated that SVs can cause major phenotypic variations that affect a series of important traits [7,39] and may complement SNP-based GWAS in identifying associations with phenotypes [47]. In the present study, we detected a lead SV associated with the abundance of the Bacteroidales order. This lead SV was located in the intron of the *DSCAM* gene, which plays an important role in immune specificity and memory [28,48]. This SV and *DSCAM* could serve as candidate markers for discovering the relationship between the host and its microbiota. In conclusion, the pangenome graph and detected SVs provide new insights for studying complex porcine traits.

Our study offers an opportunity to investigate the divergence of Y chromosomes between European and Asian pigs in detail using long-read sequencing. We identified different haplotypes in the *ZFY* gene between European and Asian pigs. In humans, this gene regulates the transcription of some Y-linked genes [49] and mediates multiple aspects of spermatogenesis and reproduction, such as capacitation, acrosome reaction, and oocyte activation [50]. The haplotype disparity of this gene between European and Asian pigs revealed the diversification of Eurasian pigs, which might be related to discrepancies in reproductive traits. In addition, a 394-kb region containing SVs exclusive to Asian pigs was discovered. This region exhibited significantly lower SNP density than the average density, making it easily omitted in SNP-based analyses. In this region, we detected a ∼ 7-kb deletion in the intronic region (near the 5′ UTR) of the LOC110257970 gene. This gene is a BCL-6 co-repressor-like gene homologous to the human *BCOR* gene. *BCOR* plays a critical role in early embryonic development and may also be involved in specifying the left and right sides of the body in developing embryos [35]. The discovery of this unique SV in Chinese domestic pigs suggests its potential as a genetic marker for studying the differences between European and Asian pigs during embryonic development.

## Materials and methods

### DNA extraction and genome sequencing

Genomic DNA was isolated from blood samples of male Meishan, Tongcheng, and Laiwu pigs using the DNeasy Blood & Tissue Kit (Catalog No. 69504, QIAGEN, Hilden, Germany). The DNA integrity was determined using an Agilent 4200 Bioanalyzer (Agilent Technologies, Santa Clara, CA). Briefly, 8 mg of DNA was sheared using g-tubes (Catalog No. 520079, Covaris, Woburn, MA) and concentrated using AMPure PB magnetic beads (Catalog No. 100-265-900, Pacific Biosciences, Menlo Park, CA). Each SMRT bell library was constructed using a Pacific Biosciences SMRT bell Template Prep Kit (Catalog No. 100-259-100, Pacific Biosciences). The constructed libraries were size-selected on a BluePippin system (Sage Science, Beverly, MA) to isolate molecules of ∼ 20 kb, and a Sequel Binding and Internal Control Kit 3.0 (Catalog No. 101-500-400, Pacific Biosciences) was used for primer annealing and binding of the SMRT bell templates to the polymerase process. Sequencing was performed on a PacBio Sequel II platform (Pacific Biosciences) using 18 SMRT cells.

### Hi-C library construction and sequencing

Approximately 10 ml of blood drawn from each sample was used for the Hi-C experiment. Blood was first crosslinked in a 2% formaldehyde solution for 15 min, and the crosslinking reaction was stopped by adding glycine. After the nuclei were isolated, the chromatin was digested with *Mbo*I. The sticky ends of the digested fragments were biotinylated, diluted, and ligated randomly. The DNA fragments labeled with biotin were sheared by ultrasound, blunt-end repaired, and A-tailed. The adapters were then ligated to the DNA fragments, and PCR amplification was performed to construct the Hi-C library. After quality control, the Hi-C library was sequenced using an Illumina paired-end sequencing platform with 2 × 150 bp reads.

### RNA-seq

RNA-seq was performed on 37 tissue samples from the liver, spleen, kidney, lung, thymus gland, stomach, duodenum, lymph, ovary, and muscle isolated from three individuals. Total RNA was extracted from each tissue sample using a TRIzol-based RNA extraction kit (Catalog No. 15596026CN, Invitrogen, Carlsbad, CA). RNA degradation and contamination were monitored using 1% agarose gel electrophoresis. The concentration of total RNA was measured using a Qubit RNA Assay Kit on a Qubit 2.0 Fluorometer (Catalog No. Q32852, Life Technologies, Carlsbad, CA). RNA-seq libraries with 250–350 bp insert sizes were prepared using QIAseq Stranded Total RNA Kit (Catalog No. 180450, QIAGEN). All libraries were sequenced on the Illumina NovaSeq 6000 S4 platform according to the manufacturer’s instructions to obtain transcriptome profiles.

### Genome assembly

All subreads from PacBio sequencing for each breed were assembled using Falcon (v2018.03.12) [51]. The assembled genomes were then polished using Pilon (v1.23) [52] with the filtered Illumina paired-end reads described above. Several rounds of iterative error correction were performed to ensure the accuracy of the genomes. Reads from the Hi-C library were then used to construct pseudo-chromosomes. After removing the adapter sequences and low-quality bases, these reads were aligned to the corresponding assembly using the aln and sample commands from bwa (v0.7.17). The resulting BAM files and contigs from the assembly were used as inputs for LACHESIS (https://github.com/shendurelab/LACHESIS) with the cluster number set to 20 and anchored to the pseudo-chromosomes. Finally, chromosome-level genomes were manually optimized using JuiceBox (v2.20.00) [53].

### Genome assembly assessment and annotation

Our assembled genomes and the other 16 public genomes used in this study were assessed using BUSCO (v5.0.0) [21] based on the lineage dataset vertebrata_odb10 (creation date: 2019-11-20). RepeatMasker (v4.1.2) (http://www.repeatmasker.org) was used to detect repeats in genomes. Gene prediction was conducted by combining three independent approaches in each repeat-masked genome, including *ab initio* prediction, homology-based prediction, and transcriptome-based prediction. For *ab initio* gene prediction, BRAKER2 (v2.1.6) [54] and GlimmerHMM (v3.0.4) were used with default parameters. For homology-based prediction, protein sequences from humans (*Homo sapiens*), mice (*Mus musculus*), cows (*Bos taurus*), sheep (*Ovis aries*), and Sscrofa11.1 reference pig genome (*Sus scrofa*) were supplied, and the gene models were predicted by GeMoMa (v1.9) [55]. For transcriptome-based prediction, the RNA-seq data of each breed were aligned to the corresponding assembly by HISAT2 (v2.2.1) with default parameters. StringTie (v2.1.6) and TransDecoder (v5.5.0, https://github.com/TransDecoder/TransDecoder) were then used to assemble the transcripts and convert candidate coding regions into gene models. Simultaneously, these RNA-seq data were also *de novo* assembled by Trinity (v2.1.1), and PASA (v2.5.3) was utilized to predict the gene structure. Finally, the gene models predicted through the abovementioned three approaches were combined by EvidenceModeler (v2.1.0) [56] into a non-redundant set of gene structures.

### Core and dispensable gene family clustering

The core and dispensable gene sets were estimated based on gene family clustering using OrthoFinder (v2.5.4) [57]. For each genome, a gene whose CDS was 100% similar to that of the other genes was removed using the cd-hit-est function of CD-HIT (v4.8.1) [58]. The protein sequences of the remaining genes were processed using OrthoFinder with diamond (v2.0.7.145). Based on the results, gene families were categorized into four classes: core, softcore, dispensable, and private gene families. We used InterProScan (v5.47-82.0) [59] to annotate the protein domain using Pfam and GO datasets.

### SNP analysis of different breeds

The remaining 18 genomes were aligned to the reference genome Sscrofa11.1 using Mummer (v4.0.0rc1) [60] with the parameter settings “-g 1000 -c 90 -l 40”. The alignment block was then filtered out of the mapping noise, and the one-to-one alignment was identified using a delta-filter with the parameter setting “-1”. Show-snps was used to identify SNPs with the parameter setting “-ClrT”. All clean reads for the 599 pigs across Eurasian were mapped to the Sscrofa11.1 genome using the “MEM” function of bwa. SAMtools (v1.15) was used to sort the mapped reads, and samblaster (v.0.1.26) was applied to mark potential PCR duplications. The GATK (4.1.2.0) HaplotypeCaller best practice was used to detect SNPs. Obtained SNPs were filtered using the VariationFiltration in GATK, according to the following criteria: (1) approximate read depth > 10×; (2) variant confidence/quality by depth > 2.0; (3) RMS mapping quality (MQ) > 40.0; (4) Phred-scaled *P* value using Fisher’s exact test to detect strand bias < 60.0; (5) Z-score from the Wilcoxon rank sum test of Alt *vs*. Ref read MQs (MQRankSum) > −12.5; and (6) Z-score from the Wilcoxon rank sum test of Alt *vs*. Ref read position bias(ReadPosRankSum) > −8.0.

### SV identification

Previously filtered Mummer alignment delta files were used to detect SVs using smartie-sv (https://github.com/zeeev/smartie-sv) with default parameters. The detected SVs from smartie-sv consisted of insertions and deletions; meanwhile, insertions/deletions were treated as the PAV region. Simultaneously, the SyRI pipeline [61] was applied based on filtered Mummer delta files with default parameters to detect inversions and translocations. According to the definitions of sequence variation in SyRI outputs, we converted the INV variants into inversion SVs relative to Sscrofa11.1. Simultaneously, TRANS and INVTR were considered translocation SVs relative to Sscrofa11.1.

### Identification of SV hotspot regions

We calculated the distribution of SV breakpoints for each 200-kb window (with a 100-kb step size) along each chromosome. Then, all 200-kb windows were ranked in descending order according to the number of SVs within the window. The top 5% of all windows with the highest frequency of SV breakpoints were regarded as SV hotspots. All consecutive hotspot windows were merged as the “hotspot regions”. Simultaneously, we employed the same method to identify the hotspot regions for each subpopulation.

### Graph-based pangenome construction and utilization

SVs from the 18 genomes were filtered using a previously reported pangenome construction pipeline [9]. In brief, redundant SVs were removed, and the remaining SVs with more than 90% repetitive sequences were omitted. The Sscrofa11.1 genome was set as a reference and combined with the non-redundant SVs to build the graph-based pangenome using vg (v1.56.0) [62]. The “Giraffe” pipeline was used for SV genotyping [63], and the resulting variant call format file for each individual was merged using VCFtools (v0.1.17) [64].

The heritability of ASVs and gene expression was estimated using a previously reported pipeline [16]. Briefly, the following genetic variants were removed: (1) mean depth < 5×, (2) missing rate > 95%, and (3) minor allele frequency < 0.1. For SNPs, we performed LD-based pruning using PLINK (v.1.9) [65] with the “--indep-pairwise 50 10 0.1” option, while the LD-based pruning was not performed on SVs. The LDAK-thin model [27] was applied to estimate the proportion of phenotypic variance explained by genetic variants. Finally, we added the parameter “--constraint YES” to ensure that all estimations with LDAK-thin are bounded within [0,1].

We performed GWAS using a mixed linear model (MLM) method. Furthermore, we used the leave-one-chromosome-out (LOCO) method and performed association studies implemented in GCTA (v1.93.2beta) [66]. Genetic variants identified in the heritability analysis were incorporated into the genetic relationship matrix (GRM). A constant threshold of 1 × 10^−6^ was used as the significance threshold. The eQTL analysis was performed by MatrixEQTL (v2.3).

### Detection of selective sweeps

For SVs, we tested the selective sweeps between European commercial pigs and Asian indigenous pigs using the *V*_ST_ approach. *V*_ST_ [33], a static analog of *F*_ST_, estimates population differentiation based on quantitative intensity data and varies from 0 to 1. The statistical *V*_ST_ of each SV was calculated to determine the selective signals between the European and Asian domestic pigs. *V*_ST_ was calculated as follows:


$$V_{\mathrm{ST}}=\frac{V_{T}-V_{S}}{V_{T}}$$

where *V*_T_ is the variance of SVs among all unrelated individuals in the target and control populations, and *V*_S_ is the average variance within each population, which was weighted for population size. SVs with the top 1% *V*_ST_ values were considered under significant selection.

We scanned the selective signals for SNPs using *F*_ST_ and nucleotide diversity (π) values. The *F*_ST_ and π values were calculated using VCFtools with sliding windows, performed with a 100-kb window size and 10-kb step size. Selective sweeps identified by SNPs were determined by merging continuous genomic regions displaying the top 5% *F*_ST_ and π (European pigs/Asian pigs) values.

### Paternal analysis and NUMT detection

We aligned the long reads of five males to the Sscrofa11.1 reference genome using minimap2 (v 2.17) and applied sniffles (v 2.0.7) to detect SVs on chromosome Y. The NUMTs in the 19 assemblies were detected using a previously reported method [36]. In brief, two copies of the mitochondrial sequence from Sscrofa11.1 (AF486858.1) were concatenated and then compared to the genome assemblies using GABLAM [67], which is a wrapper for BLAST+ to identify potential NUMTs. The age of each NUMT was estimated using a previously described method [68]. Briefly, the mitochondrial sequences from Sscrofa11.1 and the common warthog (*Phacochoerus africanus*, DQ409327.1) were aligned with the consensus sequence of each NUMT contig using MUSCLE (v3.8.31) [69]. We then quantified the number of sites within each NUMT region where modern and ancestral mitochondrial sequences differed. The ratio of sites matching the modern allele to all different sites was calculated and used to derive an approximate age for each NUMT relative to the estimated divergence time of 9.7 million years between *Sus scrofa* and *Phacochoerus africanus* [70]. To ensure the accuracy of the results, we only retained NUMTs with sizes between 50 and 1000 bp and excluded NUMTs without different alleles between Sscrofa11.1 and the common warthog.

### SV validation

The SVs under selection located in the CDS and promoter regions were validated using specific PCR assays for the 13 distinct Eurasian pig breeds. Primers were designed for each SV using Primer3 (v4.0.1) based on sequences around the breakpoints in Sscrofa11.1. The PCR amplifications were performed in 25 μl reaction volumes using the 2×EasyTaq PCR SuperMix (Catalog No. AS111-11, Trans, Beijing, China) and processed as follows: initial denaturation at 94°C for 5 min; 32 cycles of amplification (94°C for 30 s, 58°C for 30 s, and 72°C for 1 min); and a final extension at 72°C for 5 min.

### Luciferase reporter assay of the *TBX19* promoter region

The full-length promoter of *TBX19*, with or without the insertion, was chemically synthesized. Then, the two types of *TBX19* promoter regions were cloned into the pGL3-Basic luciferase vector (Jinkairui, Wuhan, China) using the *Nhe*I/*Xho*I restriction sites (Jinkairui). Sequencing was performed to verify the plasmid identities. The recombinant plasmids, along with the pRL-TK plasmid (Jinkairui) as an internal control, were transfected into the 293T cells using Lipofectamine 2000 (Catalog No. 11668-019, Invitrogen, Carlsbad, CA). After 48 h of incubation, firefly and Renilla luciferase activities were quantified using the Dual-Luciferase Reporter Assay System (Catalog No. 11402ES60, Yeasen Biotechnology, Shanghai, China).

## Ethical statement

This study was conducted in strict accordance with the protocol approved by the Institutional Animal Care and Use Committee (IACUC) at the China Agricultural University (Approval No. DK996).

## Code availability

The code used in the study is available at GitHub (https://github.com/kimi-du-bio/GBPPG). The code has also been submitted to BioCode at the National Genomics Data Center (NGDC), Beijing Institute of Genomics (BIG), Chinese Academy of Sciences (CAS) / China National Center for Bioinformation (CNCB) (BioCode: BT007579), which is publicly accessible at https://ngdc.cncb.ac.cn/biocode/tool/BT007579.

## Supplementary Material

qzae081_Supplementary_Data

## Data Availability

The raw sequencing data generated in this study have been deposited in the Genome Sequence Archive [71] at the NGDC, BIG, CAS / CNCB (GSA: CRA012558), and are publicly accessible at https://ngdc.cncb.ac.cn/gsa. The three chromosomal-level genome assemblies of Chinese native pigs have been deposited in the Genome Warehouse [72] at the NGDC, BIG, CAS / CNCB (GWH: GWHDTXD00000000 for Laiwu pigs, GWHDTXE00000000 for Meishan pigs, and GWHDTXM00000000 for Tongcheng pigs), and are publicly accessible at https://ngdc.cncb.ac.cn/gwh. The SNPs and SVs detected in this study have been deposited in the Genome Variation Map [73] at the NGDC, BIG, CAS / CNCB (GVM: GVM000861), and are publicly accessible at https://ngdc.cncb.ac.cn/gvm. The genome annotation results and graph-based pangenome generated in this study are publicly accessible at Zenodo (https://doi.org/10.5281/zenodo.12794748).

## CRediT author statement

**Heng Du:** Investigation, Writing – original draft. **Yue Zhuo:** Investigation, Writing – original draft. **Shiyu Lu:** Validation. **Wanying Li:** Validation, Writing – review & editing. **Lei Zhou:** Writing – review & editing. **Feizhou Sun:** Resources, Writing – review & editing. **Gang Liu:** Resources. **Jian-Feng Liu:** Conceptualization, Methodology, Supervision, Writing – review & editing. All authors have read and approved the final manuscript.
