# Supplementary material for: Pangenome Reveals Gene Content Variations and Structural Variants Contributing to Pig Characteristics
Source: Genomics Proteomics Bioinformatics. 2024 Nov 13;22(6):qzae081. doi: 10.1093/gpbjnl/qzae081 (PMC12017589; doi:10.1093/gpbjnl/qzae081)
Supplement: qzae081_Supplementary_Data [file qzae081_supplementary_data.zip › supplementary material captions.docx]

**Supplementary materials**

**File S1 Supplementary information**

**Figure S1 The neighbor-joining tree was constructed by 599 genomes**

**Figure S2 The frequency distribution of *k*-mer for each genome (*k* = 17)**

**A.** Laiwu pig. **B.** Meishan pig. **C.** Tongcheng pig.

**Figure S3 Genome-wide contact matrix of three pig genomes**

**A.** Tongcheng pig. **B.** Laiwu pig. **C.** Meishan pig. The color intensities in these three plots represent the frequency of contact between two 500 kb loci.

**Figure S4 The SNPs were identified by assembly-comparison for each assembly**

**Figure S5 Correlation of SNP density, π, Ts/Tv, and Tajima'd from 18 *de novo* assembled genomes (compared to the reference genome) and 599 resequencing samples**

**A.** SNP density (number of variants in 1kb region). **B.** π. **C.** Ts/Tv. **D.** Tajima'd.

**Figure S6 Characterization of SVs in the 18 pig genomes**

**A.** Absence. **B.** Presence. **C.** Inversion. **D.** Translocation. The heatmaps in the four figures show the frequency of SVs in 18 genomes.

**Figure S7 Chromosome distributions of hotspot SVs in European pig population, BL, DWB, RT, and TNJM populations**

The red box indicates the region with hotspots that have not been detected in the European population, while the blue box indicates that only the European population detects the hotspots.

**Figure S8 The annotation results of PAVs with genes and their flanking regions**

**Figure S9 The pangenome graph shows variations within 2175210-2175820 bp on chromosome 1**

**Figure S10 The GWAS result of ASV2205**

**A.** The Manhattan plot. **B.** The quantile-quantile plot.

**Figure S11 The RNA-Seq alignment results of *RSAD2***

**Figure S12 The genomic regions under selection are inferred by SNPs**

**A.** *F*_ST_ results. **B.** Nucleotide diversity result. **C.** The SNPs are selected by two methods; the red dot represents the selected regions. **D.** The enrichment analysis of SVs under selection and the regions under selection detected by SNPs.

**Figure S13 The gene structure of the *LOC100624149* gene and the insertion in its exonic region**

The red arrow represents the insertion position.

**Figure S14 The gene structure of the *LOC110257970* gene and the deletion in its intronic region**

The blue rectangle represents the deletion region.

**Figure S15 The NUMTs positions are distributed in all chromosomes**

**Table S1 The 599 high-throughput Sequencing data used in this study**

**Table S2 The pig genomes were collected from the previous studies**

**Table S3 The detailed information for Illumina reads, PacBio reads, and Hi-C reads**

**Table S4 Statistics of assembled contigs for three pig genomes**

**Table S5 Profile of the annotated genes in the three new genomes**

**Table S6 Evaluation of protein completeness for three new assemblies using BUSCO**

**Table S7 Statistics profile of gene function annotation for new three genomes**

**Table S8 The RNA-Seq data of 171 samples were used in this study**

**Table S9 The 495 hotspots and related QTLs**

**Table S10 ASVs assigned taxonomy**

**Table S11 The SVs have the high LD with the tag SNPs**

**Table S12 The enriched KEGG pathways for genes are influenced by SVs under significant selection**

**Table S13 1005 QTLs overlapped with the genes influenced by SVs**

**Table S14 Nine genes in which CDS was influenced by SVs**

**Table S15 The enriched GO terms for genes whose promoters were influenced by SVs under significant selection**

**Table S16 The five SVs in the 394 kb region of chrY and their overlapped genes**

**Table S17 The genes overlapped with NUMTs**

**Table S18 The primers used in this study**
