# Supplementary material for: Pangenome Reveals Gene Content Variations and Structural Variants Contributing to Pig Characteristics
Source: Genomics Proteomics Bioinformatics. 2024 Nov 13;22(6):qzae081. doi: 10.1093/gpbjnl/qzae081 (PMC12017589; doi:10.1093/gpbjnl/qzae081)
Supplement: qzae081_Supplementary_Data [file qzae081_supplementary_data.zip › File S1.docx]

**Supplementary matetial**

**Genome size estimation**

Before *de novo* assembly, we estimated the genome size of the three breeds. For each breed, an Illumina short-read library was constructed using DNA from an identical PacBio sequencing source. More than 160 Gb ( > 60×) of 150-bp paired-end reads were generated for each breed. Adapter sequences and low-quality reads (base quality [Q] values < 20) were removed and trimmed using TrimGalore (v0.6.1) [1]. Based on the remaining data, Jellyfish (v2.3.0) [2] was used to estimate the genome using the *k*-mer method. The length of each *k*-mer was set to 17 base pairs (bp). A clear peak depth could be distinguished from the *k*-mer depth distribution computed using Jellyfish (Figure S1). The genome size of each breed was then calculated using the following formula: Genome size=K-num/K-depth, where K-num is the total number of *k*-mers, and K-depth is the highest *k*-mer frequency.

**The annotation of genomic variations and enrichment analysis**

The ANNOVAR (2020-06-07) [3] was used to annotate the genomic variations based on the Ensembl dataset (Release 112). The enrichment analysis of genes was conducted by KOBAS-I [4].

**The population structure analysis conducted by SNPs**

We constructed a neighbour-joining (NJ) tree using the SNPs detected in 599 pigs across Eurasia. Pairwise genetic distances were first calculated using emmax (beta-07Mar2010) [5] based on these SNPs, and then MEGA (v11) [6] was used to construct the NJ tree.

**The gene expression profile analysis**

The RNA-Seq data was aligned to the Sscrofa11.1 using HISAT2 (v2.2.1) [7]. The alignment results were assembled by StringTie (v2.1.6) [8], and the script in-house calculated the TPM.

**The mechanism prediction for SV formation**

We used BreakSeq (v1.3) [9] to predict the formation mechanism for absences, presences and inversions.

**The enrichment analysis of SVs and different genomic features**

We conducted an enrichment analysis to assess the significance of associations between the SVs and seven distinct genome features: genes, exons, introns, coding sequences (CDS), promoters, untranslated regions (UTR), and enhancers. Initially, each SV was randomly relocated within the same chromosome to establish a control group using the 'shuffle' command in the Bedtools (v2.31.0) [10] software. We then used the 'intersect' command in the Bedtools software to identify overlaps between the SVs and each of the seven genomic features, both for actual and randomized SVs. Finally, a t-test was conducted to determine whether the SVs were significantly enriched or depleted in these genomic feature regions.

**The 16s rRNA data processing**

The 16s rRNA data was downloaded from the previous study [11]. The data were processed by QIIME 2 [12] with the DADA2 [13] to create the ASV (Amplicon Sequence Variant) table.

**The performance of graph pangenome genotyping SVs**

We used the previously reported method [14] to evaluate the performance of graph pangenome with vg to genotype SVs. In brief, vg was used to simulate the short-reads with 20× coverage based on the SVs from 18 assemblies. The simulated reads were then mapped and called SVs using vg. The performance of the pangenome was evaluated using truvari (v4.2.2) [15] based on the comparison between benchmark SVs and detected SVs. The *F*_1_ score of this graph pangenome with vg reached 0.86, comparable to that of yeast [16] and soybeans [17].

**Definition of heritability category**

We defined the coordinates of three anchor dots that represented three heritability categories, including SV dominant (SNP:0, SV:1), SNP dominant (SNP:1, SV:0), and balanced (SNP:0.5, SV:0.5). The heritability was estimated based on the "SNP+SV" composite model. The heritability estimated by each type of genetic variants were used as the coordinate of each trait. The Euclidean distance between the coordinates of each trait and each anchor dot was calculated, and each trait was assigned to the category with the shortest distance.

**The SVs and SNPs on the Y chromosome of pig**

The SVs on chromosome Y were detected by sniffles based on the alignment results, which used PacBio reads aligned to Sscrofa11.1. In our collected 599 samples, 170 were male and were used to discover the SNPs on the Y chromosome by GATK. Interestingly, through scanning the SNPs in the *ZFY* gene across Eurasian pigs, we found that Asian pigs contained many SNPs in this gene, which Europeans did not. European and Asian pigs might have a distinct haplotype for this gene.

**Reference**

[1] Martin M. Cutadapt removes adapter sequences from high-throughput sequencing reads. EMBnet Journal 2011;17:10–2.

[2] Marcais G, Kingsford C. A fast, lock-free approach for efficient parallel counting of occurrences of k-mers. Bioinformatics 2011;27:764–70.

[3] Wang K, Li MY, Hakonarson H. ANNOVAR: functional annotation of genetic variants from high-throughput sequencing data. Nucleic Acids Res 2010;38:e164.

[4] Bu DC, Luo HT, Huo PP, Wang ZH, Zhang S, He ZH, et al. KOBAS-i: intelligent prioritization and exploratory visualization of biological functions for gene enrichment analysis. Nucleic Acids Research 2021;49:W317–25.

[5] Kang HM, Sul JH, Service SK, Zaitlen NA, Kong SY, Freimer NB, et al. Variance component model to account for sample structure in genome-wide association studies. Nat Genet 2010;42:348–54.

[6] Tamura K, Stecher G, Kumar S. MEGA11: molecular evolutionary genetics analysis version 11. Mol Biol Evol 2021;38:3022–7.

[7] Kim D, Paggi JM, Park C, Bennett C, Salzberg SL. Graph-based genome alignment and genotyping with HISAT2 and HISAT-genotype. Nat Biotechnol 2019;37:907–15.

[8] Pertea M, Pertea GM, Antonescu CM, Chang T-C, Mendell JT, Salzberg SL. StringTie enables improved reconstruction of a transcriptome from RNA-seq reads. Nat Biotechnol 2015;33:290–5.

[9] Lam HYK, Mu XJ, Stutz AM, Tanzer A, Cayting PD, Snyder M, et al. Nucleotide-resolution analysis of structural variants using BreakSeq and a breakpoint library. Nat Biotechnol 2010;28:47–55.

[10] Quinlan AR, Hall IM. BEDTools: a flexible suite of utilities for comparing genomic features. Bioinformatics 2010;26:841–2.

[11] Yang H, Wu J, Huang X, Zhou Y, Zhang Y, Liu M, et al. ABO genotype alters the gut microbiota by regulating GalNAc levels in pigs. Nature 2022;606:358–67.

[12] Bolyen E, Rideout JR, Dillon MR, Bokulich NA, Abnet CC, Al-Ghalith GA, et al. Reproducible, interactive, scalable and extensible microbiome data science using QIIME 2. Nat Biotechnol 2019;37:852–7.

[13] Callahan BJ, McMurdie PJ, Rosen MJ, Han AW, Johnson AJA, Holmes SP. DADA2: high resolution sample inference from Illumina amplicon data. Nat Methods 2016;13:581–3.

[14] Zhou Y, Zhang ZY, Bao ZG, Li HB, Lyu YQ, Zan YJ, et al. Graph pangenome captures missing heritability and empowers tomato breeding. Nature 2022;606:527–34.

[15] English AC, Menon VK, Gibbs RA, Metcalf GA, Sedlazeck FJ. Truvari: refined structural variant comparison preserves allelic diversity. Genome Biol 2022;23:271.

[16] Hickey G, Heller D, Monlong J, Sibbesen JA, Sirén J, Eizenga J, et al. Genotyping structural variants in pangenome graphs using the vg toolkit. Genome Biol 2020;21:35.

[17] Liu Y, Du H, Li P, Shen Y, Peng H, Liu S, et al. Pan-genome of wild and cultivated soybeans. Cell 2020;182:162–76.e13.
