# Supplementary figures and images for: Pangenome Reveals Gene Content Variations and Structural Variants Contributing to Pig Characteristics
Source: Genomics Proteomics Bioinformatics. 2024 Nov 13;22(6):qzae081. doi: 10.1093/gpbjnl/qzae081 (PMC12017589; doi:10.1093/gpbjnl/qzae081)

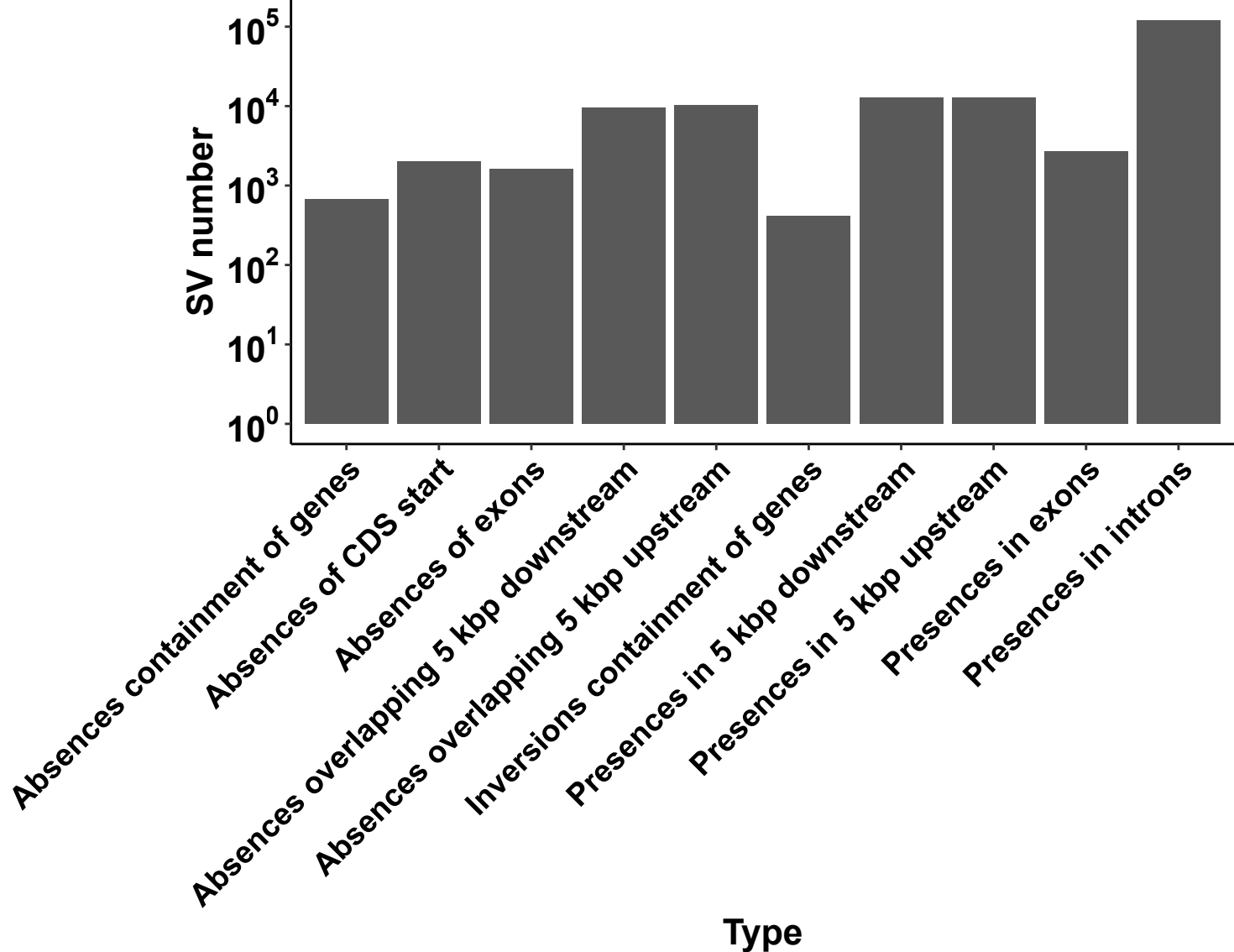

Supplement: qzae081_Supplementary_Data [file qzae081_supplementary_data.zip › Figure S8.pdf]

**A**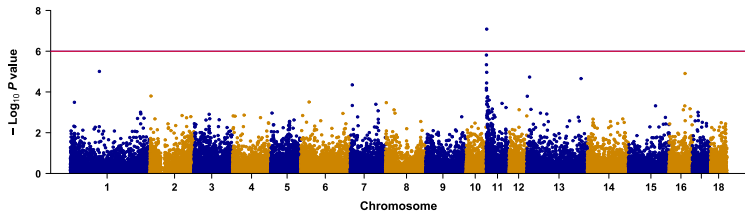**B**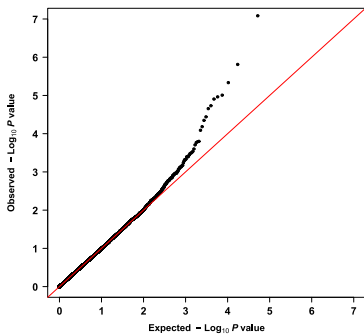

Supplement: qzae081_Supplementary_Data [file qzae081_supplementary_data.zip › Figure S10.pdf]

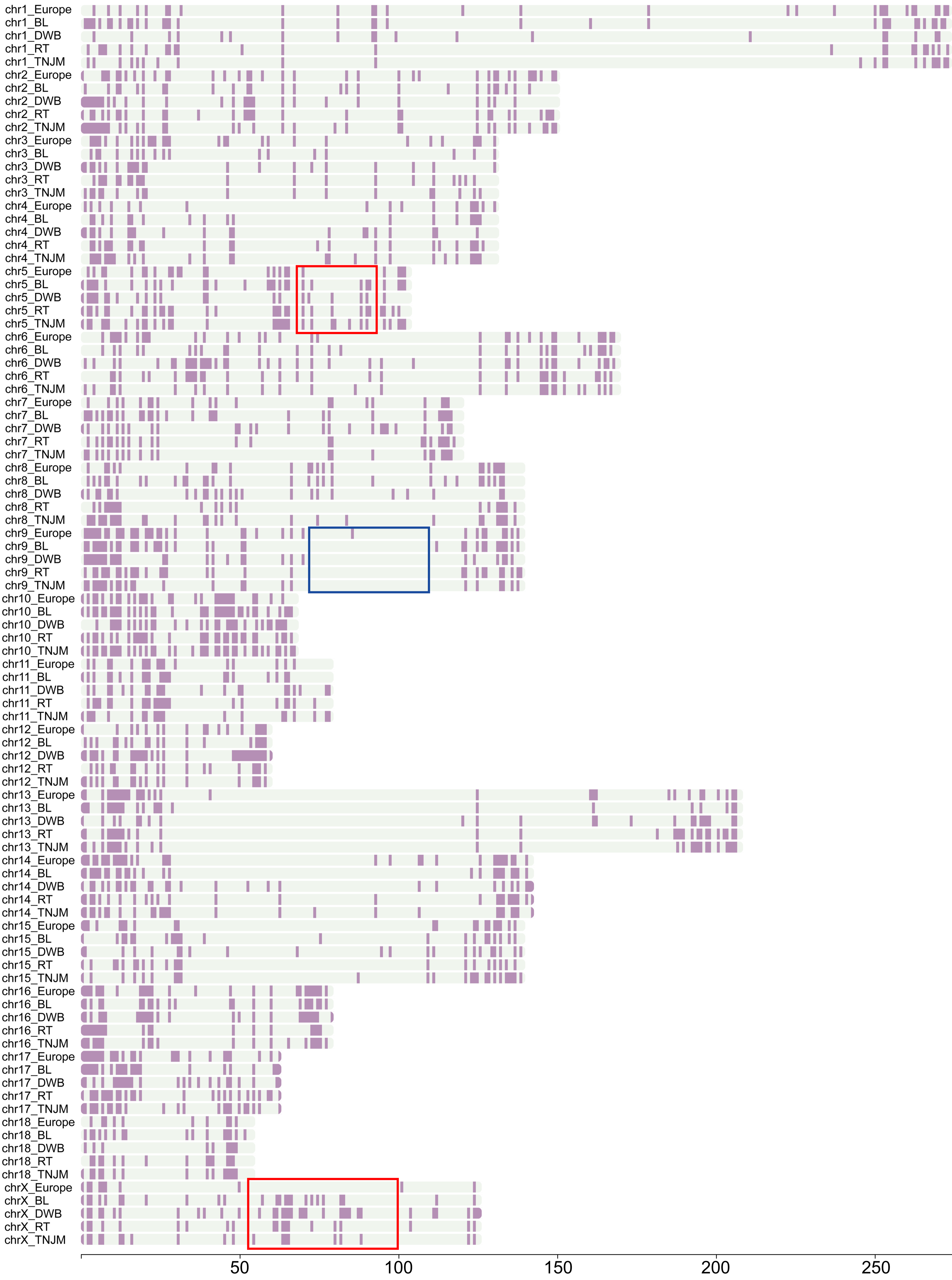

Length of chromosomes (Mb)

Supplement: qzae081_Supplementary_Data [file qzae081_supplementary_data.zip › Figure S7.pdf]

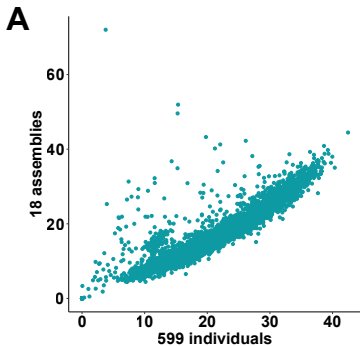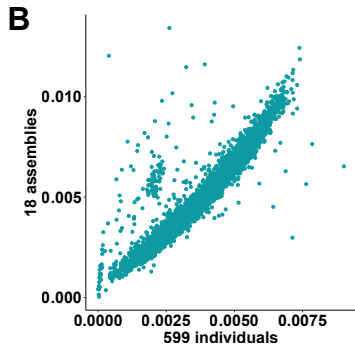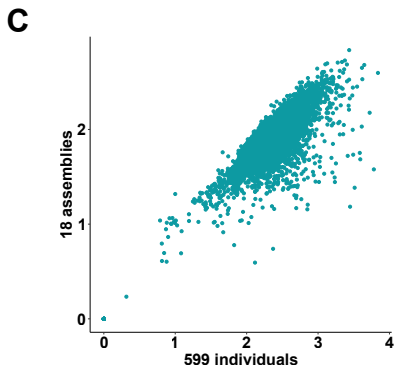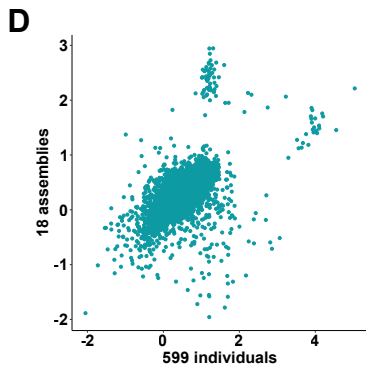

Supplement: qzae081_Supplementary_Data [file qzae081_supplementary_data.zip › Figure S5.pdf]

Tree scale: 0.01

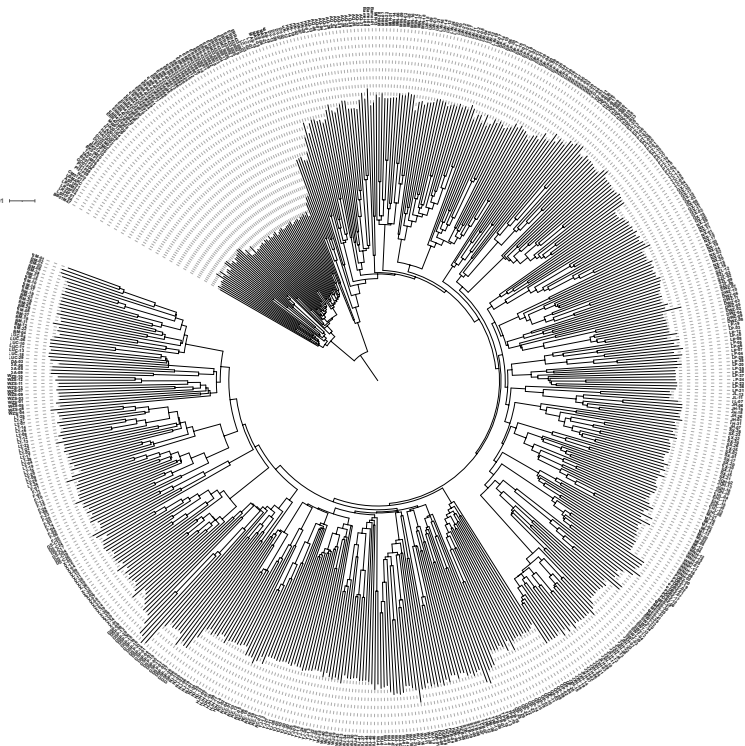

Supplement: qzae081_Supplementary_Data [file qzae081_supplementary_data.zip › Figure S1.pdf]

9.83 Mb

9.84 Mb

5'

3'

Gene

**LOC100624149**

mRNA

**LOC100624149/ENSSSCT00000013322**

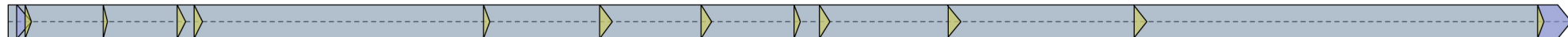

AACCCC.....AAATAAAAC(87bp)

Supplement: qzae081_Supplementary_Data [file qzae081_supplementary_data.zip › Figure S13.pdf]

**A** Tongcheng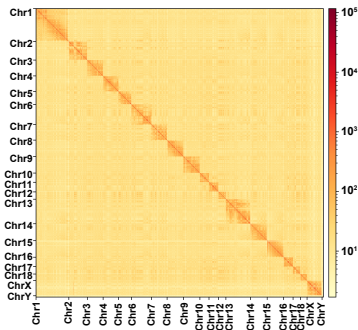**B** Laiwu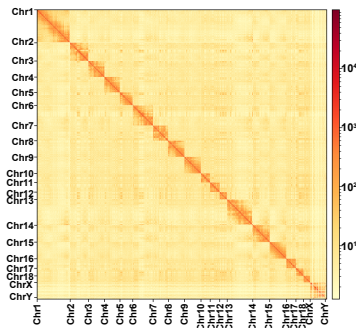**C** Meishan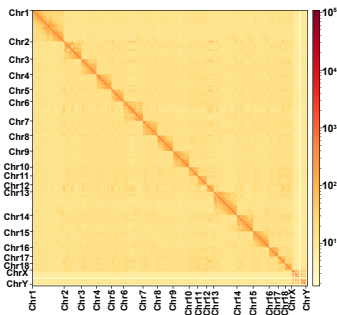

Supplement: qzae081_Supplementary_Data [file qzae081_supplementary_data.zip › Figure S3.pdf]

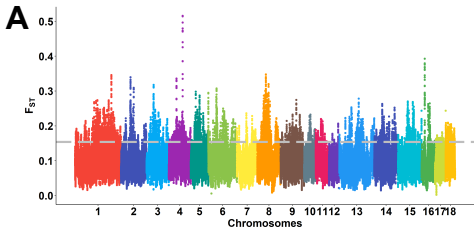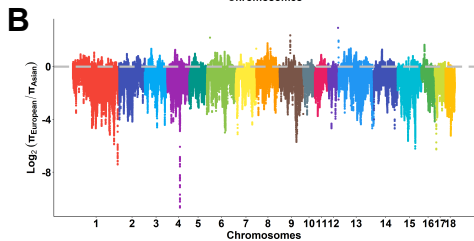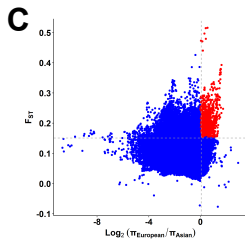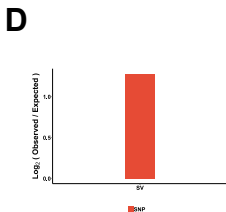

Supplement: qzae081_Supplementary_Data [file qzae081_supplementary_data.zip › Figure S12.pdf]

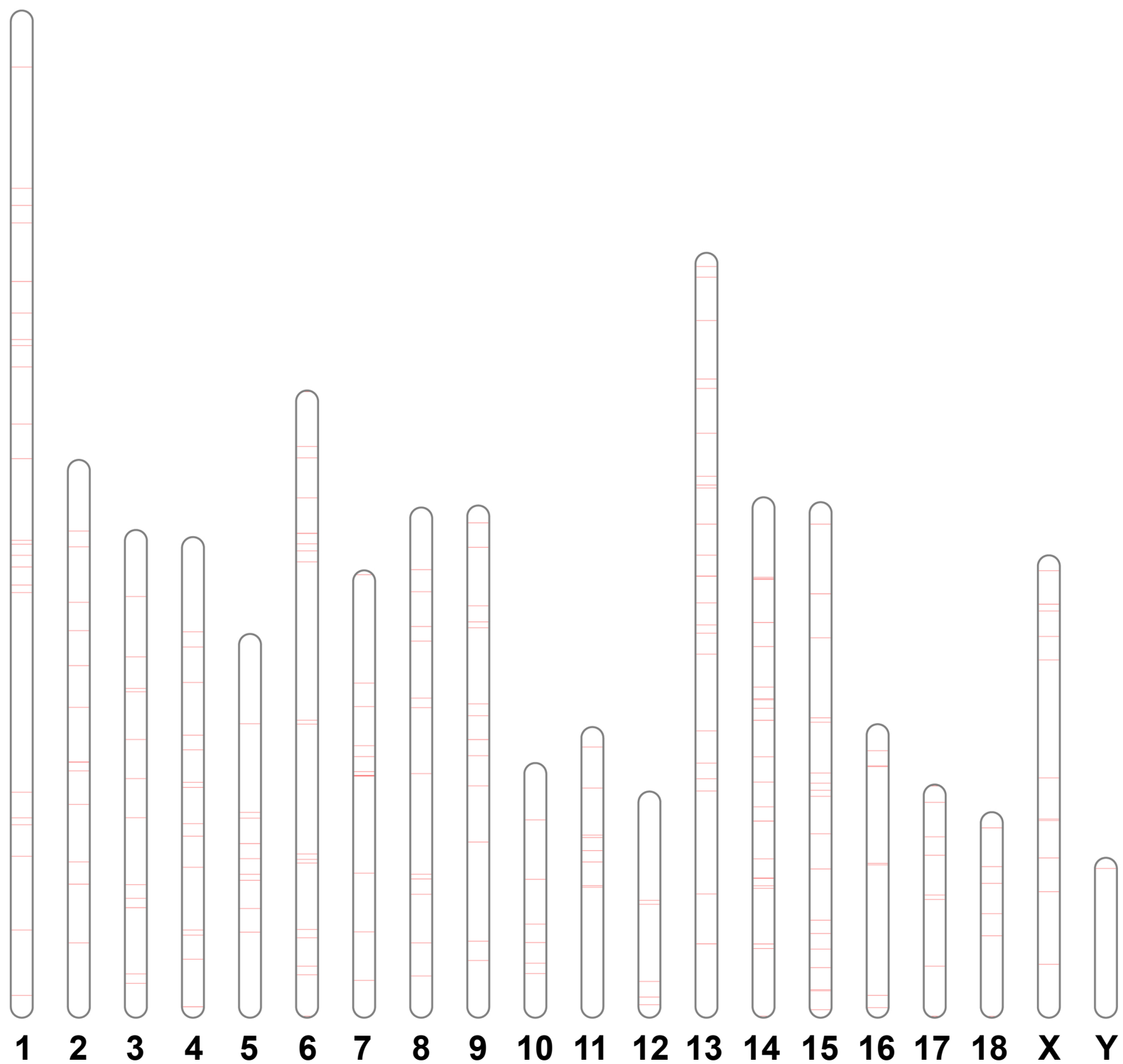

Supplement: qzae081_Supplementary_Data [file qzae081_supplementary_data.zip › Figure S15.pdf]

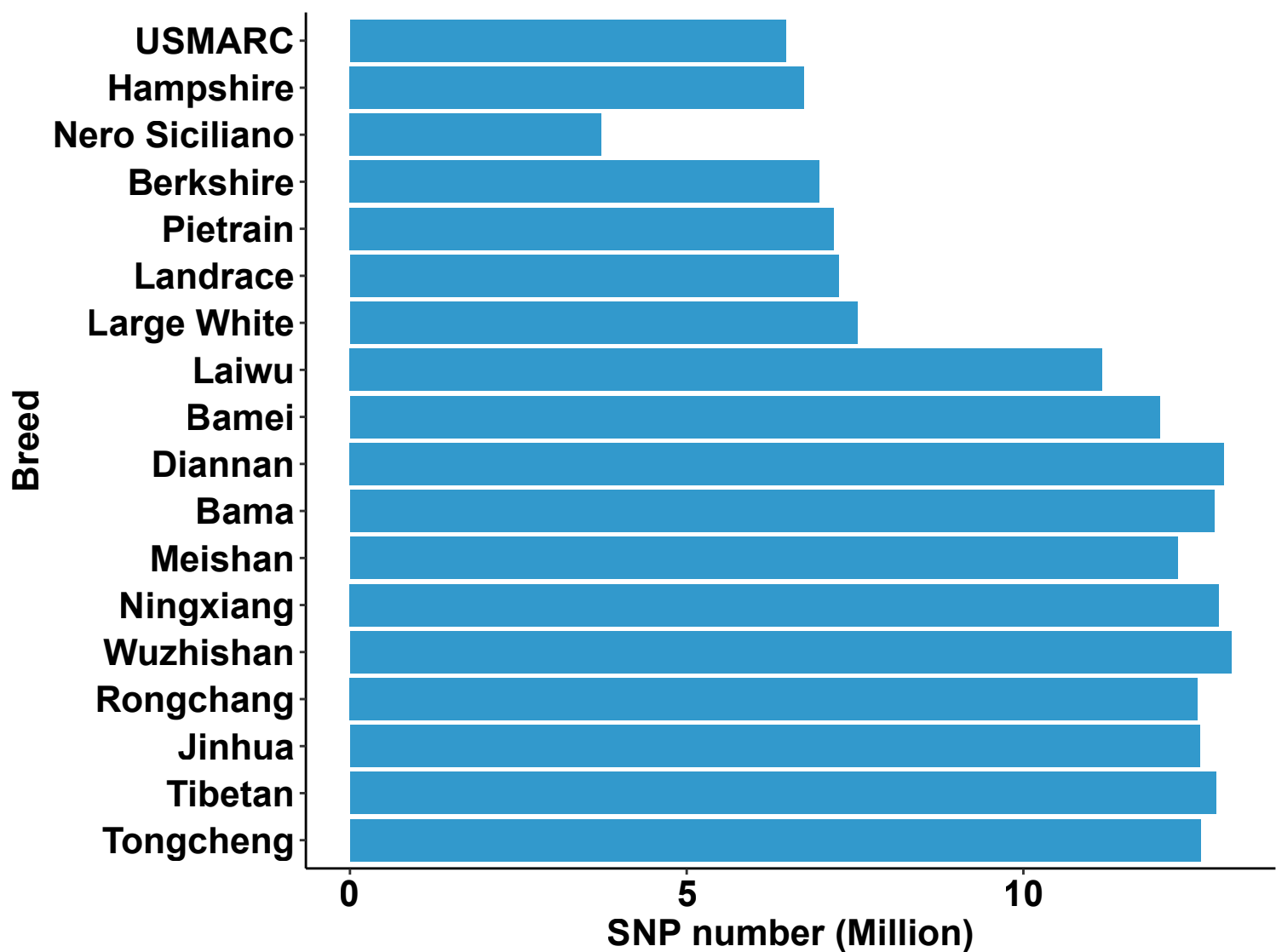

Supplement: qzae081_Supplementary_Data [file qzae081_supplementary_data.zip › Figure S4.pdf]

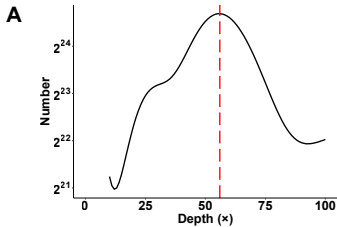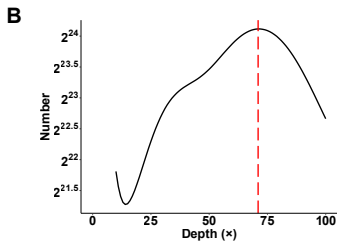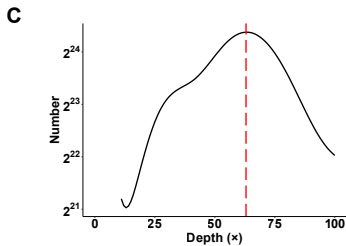

Supplement: qzae081_Supplementary_Data [file qzae081_supplementary_data.zip › Figure S2.pdf]

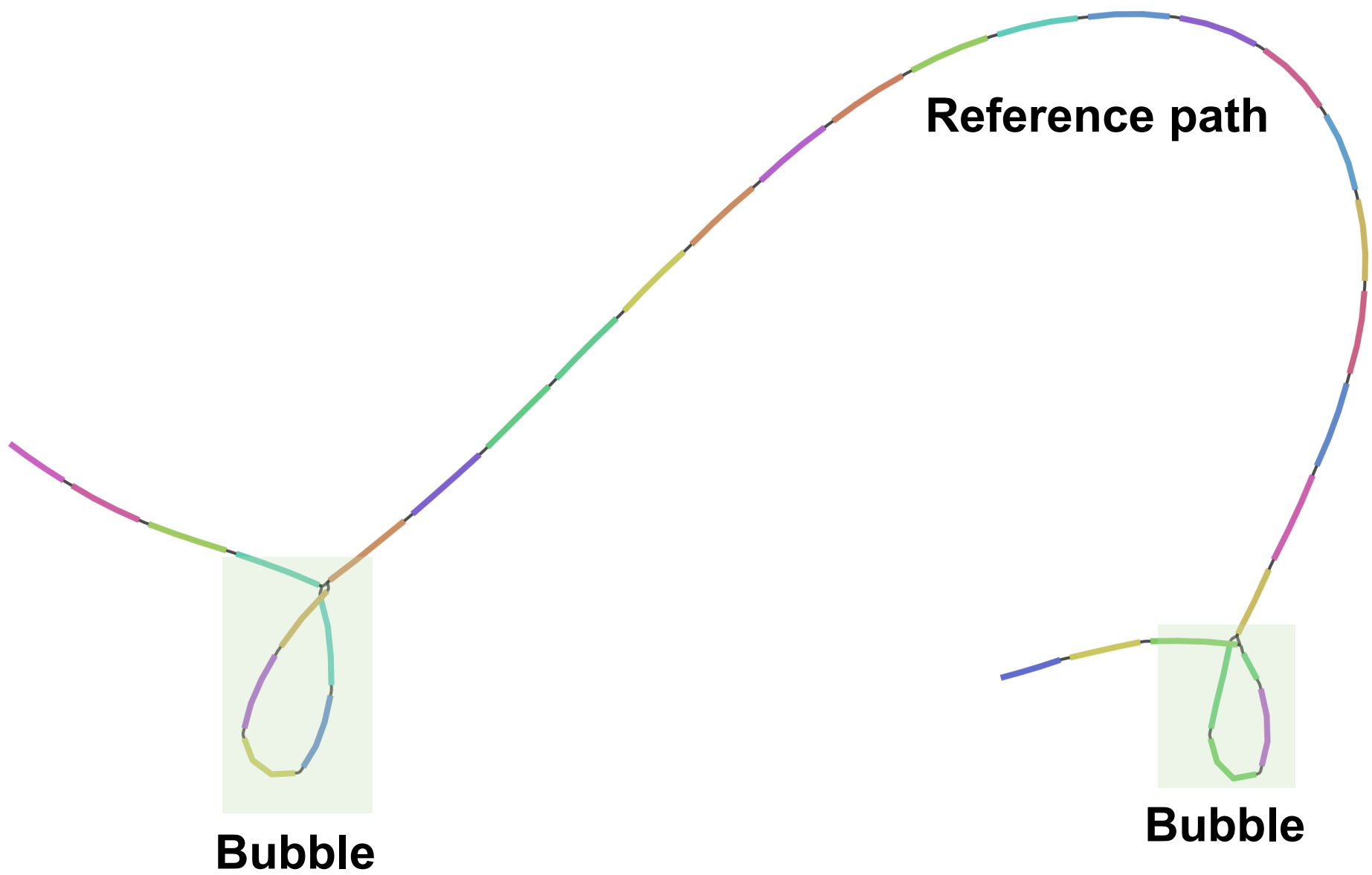

Supplement: qzae081_Supplementary_Data [file qzae081_supplementary_data.zip › Figure S9.pdf]

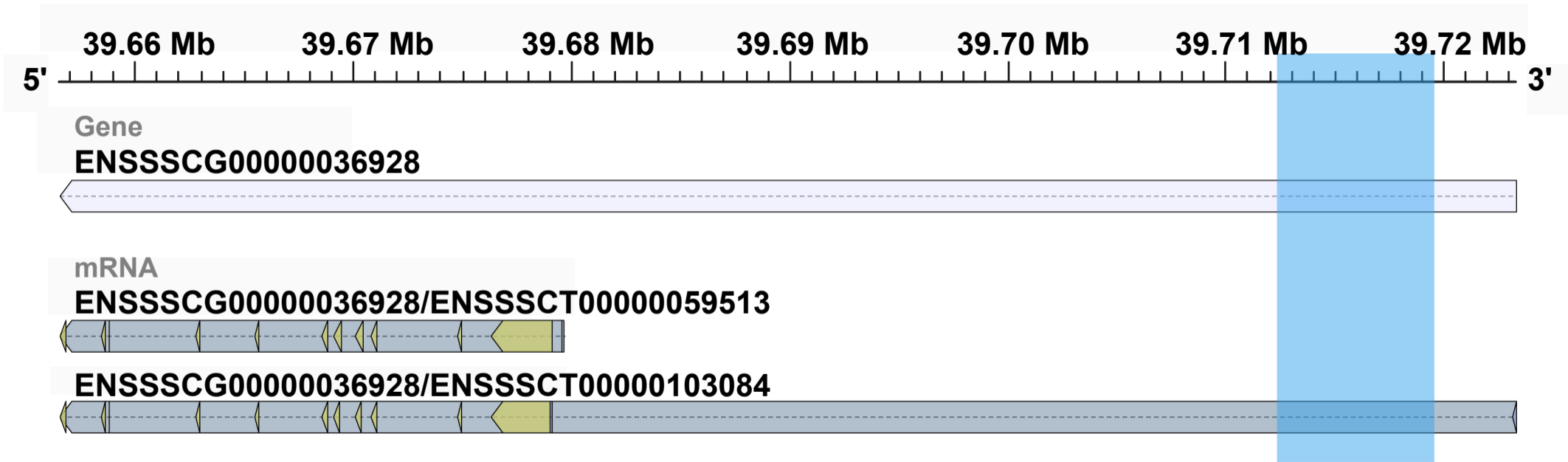

Supplement: qzae081_Supplementary_Data [file qzae081_supplementary_data.zip › Figure S14.pdf]

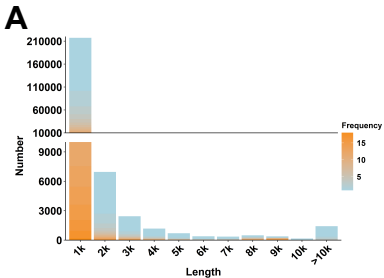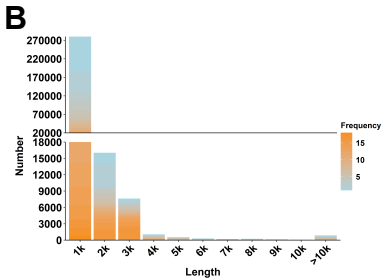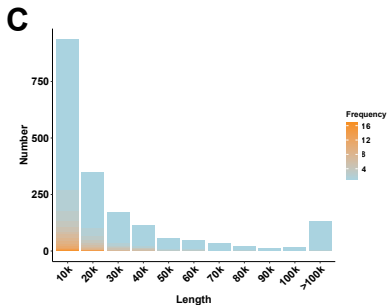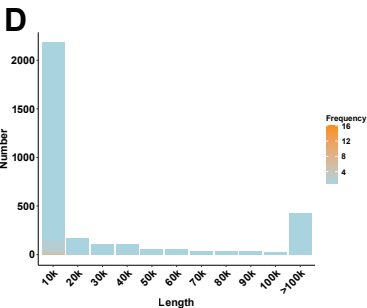

Supplement: qzae081_Supplementary_Data [file qzae081_supplementary_data.zip › Figure S6.pdf]
