# Supplementary material for: Pangenome Reveals Gene Content Variations and Structural Variants Contributing to Pig Characteristics
Source: Genomics Proteomics Bioinformatics. 2024 Nov 13;22(6):qzae081. doi: 10.1093/gpbjnl/qzae081 (PMC12017589; doi:10.1093/gpbjnl/qzae081)
Supplement: qzae081_Supplementary_Data [file Figure S11.pdf › Figure S11.pdf]

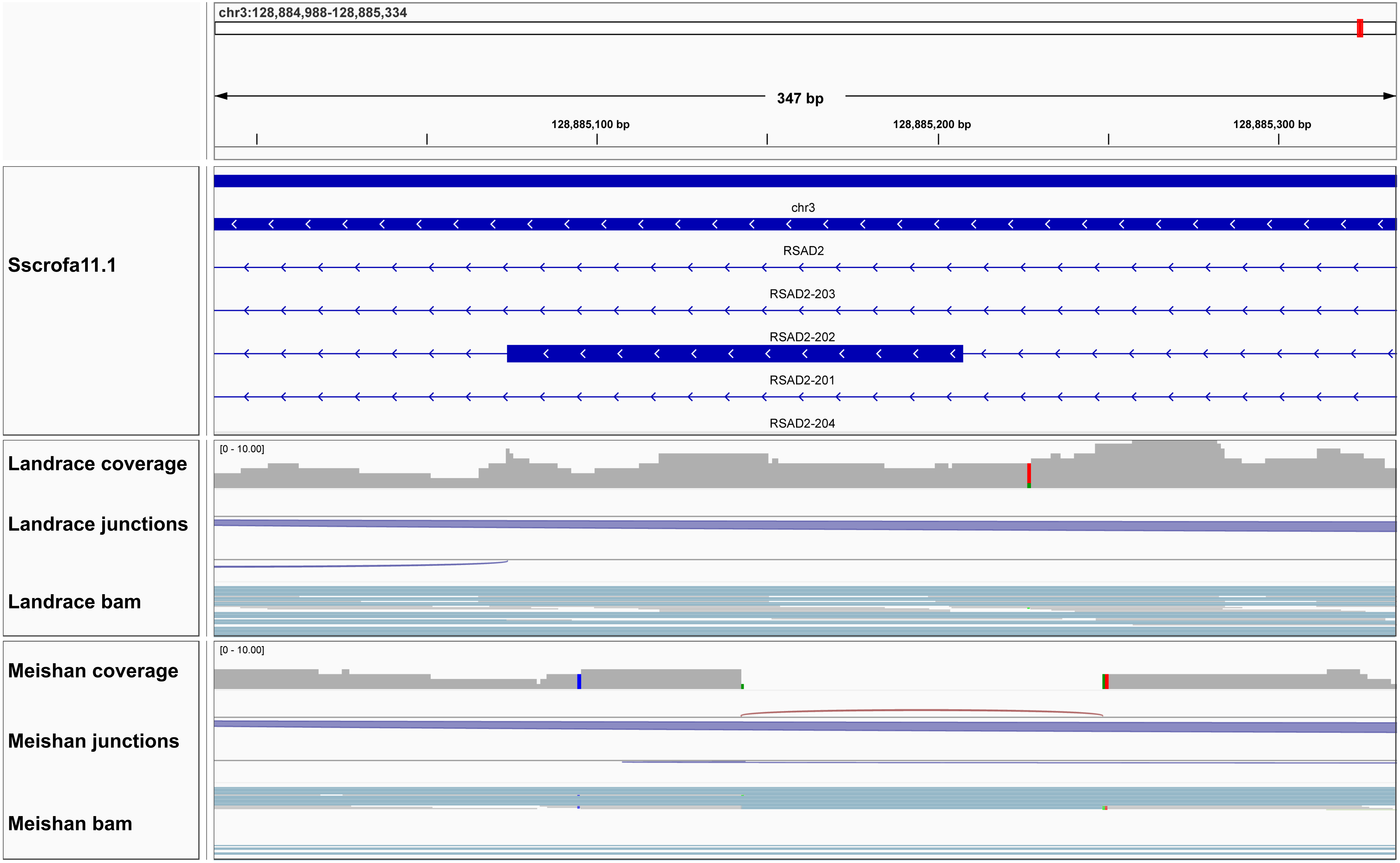
chr3:128,884,988-128,885,334
347 bp
128,885,100 bp
128,885,200 bp
128,885,300 bp
Sscrofa11.1
chr3
RSAD2
RSAD2-203
RSAD2-202
RSAD2-201
RSAD2-204
Landrace coverage
[0 - 10.00]
Landrace junctions
Landrace bam
Meishan coverage
[0 - 10.00]
Meishan junctions
Meishan bam
